# Histone H2A insufficiency causes chromosomal segregation defects due to anaphase chromosome bridge formation at rDNA repeats in fission yeast

**DOI:** 10.1038/s41598-019-43633-5

**Published:** 2019-05-09

**Authors:** Takaharu G. Yamamoto, Da-Qiao Ding, Yuki Nagahama, Yuji Chikashige, Tokuko Haraguchi, Yasushi Hiraoka

**Affiliations:** 1 Advanced ICT Research Institute Kobe, National Institute of Information and Communications Technology, 588-2 Iwaoka, Iwaoka-cho, Nishi-ku, Kobe 651-2492 Japan; 2 Graduate School of Frontier Biosciences, Osaka University, 1-3 Yamadaoka, Suita, 565-0871 Japan

**Keywords:** Chromatin, Chromosomes

## Abstract

The nucleosome, composed of DNA and a histone core, is the basic structural unit of chromatin. The fission yeast *Schizosaccharomyces pombe* has two genes of histone H2A, *hta*1^+^ and *hta*2^+^; these genes encode two protein species of histone H2A (H2Aα and H2Aβ, respectively), which differ in three amino acid residues, and only *hta2*^+^ is upregulated during meiosis. However, it is unknown whether *S. pombe* H2Aα and H2Aβ have functional differences. Therefore, in this study, we examined the possible functional differences between H2Aα and H2Aβ during meiosis in *S. pombe*. We found that deletion of *hta2*^+^, but not *hta1*^+^, causes defects in chromosome segregation and spore formation during meiosis. Meiotic defects in *hta2*^+^ deletion cells were rescued by expressing additional copies of *hta1*^+^ or by expressing *hta1*^+^ from the *hta2* promoter. This indicated that the defects were caused by insufficient amounts of histone H2A, and not by the amino acid residue differences between H2Aα and H2Aβ. Microscopic observation attributed the chromosome segregation defects to anaphase bridge formation in a chromosomal region at the repeats of ribosomal RNA genes (rDNA repeats). These results suggest that histone H2A insufficiency affects the chromatin structures of rDNA repeats, leading to chromosome missegregation in *S. pombe*.

## Introduction

In eukaryotes, genomic DNA is organized as chromatin, which comprises arrays of nucleosomes. A nucleosome contains a ~150 base pair (bp) DNA wrapped around a histone octamer, which is composed of two molecules of histones H2A, H2B, H3, and H4^1^. In addition to these canonical histones, studies have identified a variety of histone variants in metazoans, which are used differentially on the basis of the diverse functions of chromatin^2,3^. On the other hand, the chromatin of the fission yeast *Schizosaccharomyces pombe* comprises a small number of histone species: two protein species of histone H2A (H2Aα and H2Aβ), one protein species each of histones H2B, H3, and H4^4,5^, and two histone variants, Pht1 and Cnp1. Pht1 is the ortholog of histone H2A.Z^6^, and Cnp1 is the ortholog of CENP-A, the centromere-specific histone H3 variant^7^. These findings indicate that in *S. pombe*, canonical histones (H2Aα/H2Aβ, H2B, H3, and H4) and two histone variants are sufficient to achieve the broad functions of chromatin.

The *S. pombe* genome comprises multiple genes for the simple protein constituents of canonical histones: two genes (*hta1*^+^ and *hta2*^+^) for histone H2A, one gene (*htb1*^+^) for histone H2B, three genes (*hht1*^+^, *hht2*^+^, and *hht*3^+^) for histone H3, and three genes (*hhf1*^+^, *hhf*2^+^, and *hhf3*^+^) for histone H4^4,5^. *hta*1^+^ and *hta*2^+^ encode two protein species of histone H2A, H2Aα and H2Aβ, respectively, which differ in three amino acid residues. *hht1*^+^, *hht2*^+^, and *hht*3^+^ encode histone H3 with an identical amino acid residue. *hhf1*^+^, *hhf2*^+^, and *hhf3*^+^ encode histone H4 with an identical amino acid residue. Therefore, in *S. pombe*, histone H2A uniquely has a variant set of proteins among the canonical histone proteins.

It is unknown whether *S. pombe* H2Aα and H2Aβ have functional differences. As mentioned before, *S. pombe* H2Aα and H2Aβ differ in three amino acid residues. However, both bear the C-terminal stretch characteristic of metazoan H2A.X containing the serine residue S128/S127, which is phosphorylated by ataxia telangiectasia mutated/ataxia telangiectasia and Rad3-related (ATM/ATR) kinases and is required as a DNA damage checkpoint and for DNA damage repair^8^. H2Aα and H2Aβ also share another C-terminal serine residue, S121, which is phosphorylated by Bub1 kinase and is required for recruiting shugoshin proteins^9^ and the N-terminal region required for condensin binding^10^. Therefore, no obvious functional differences between H2Aα and H2Aβ have been reported in vegetative cell cycles, although studies have reported that *hta2* expression levels are up-regulated but *hta1* expression levels remain low during meiosis^11^. Therefore, in this study, we examined the possible functional differences between H2Aα and H2Aβ during meiosis in *S. pombe*.

## Results

### *hta2*^+^ deletion causes meiotic defects

**Figure 1:**
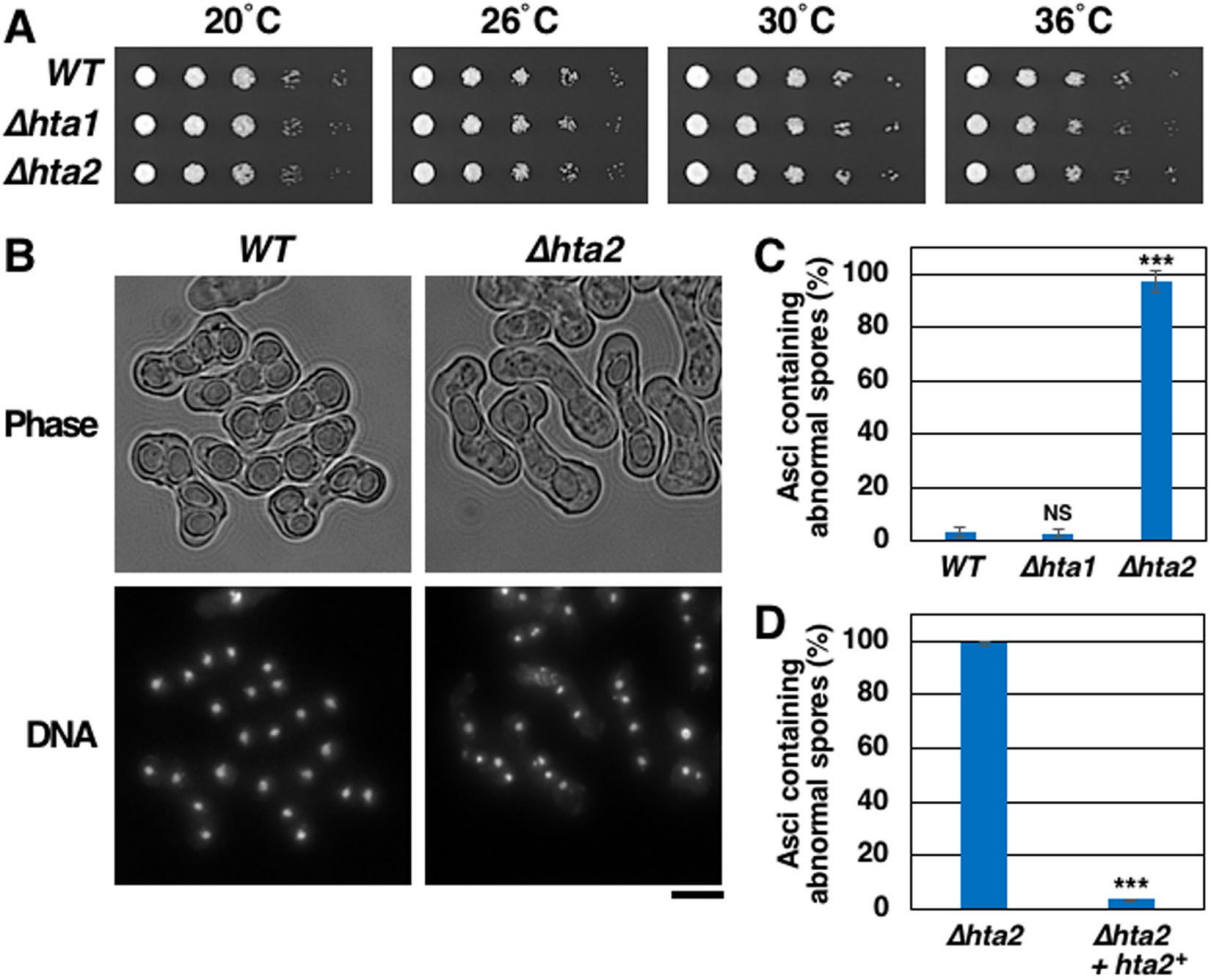
∆*hta2* cells form abnormal spores. (**A**) Spot assay comparing the growth of WT (TGO350), *∆hta1* (TGO351), and *∆hta2* (TGO352) cells. Dilution series (1/5 dilution) of cell suspensions were spotted on complete YES medium and grown for 11 days at 20 °C, 4 days at 26 °C, 3 days at 30 °C, or 3 days at 36 °C. (**B**) Abnormal spore formation in *∆hta2* cells. Meiosis was induced in WT (TGO350) and *∆hta2* (TGO352) cells, and the cells were fixed 2 days later. DNA was stained with 4′,6-diamidino-2-phenylindole (DAPI). Bright-field images of the same cells are also shown (“Phase”). Scale bar, 5 µm. (**C**) Frequency of asci containing abnormal spores in WT (TGO350), *∆hta1* (TGO351), and *∆hta2* (TGO352) cells. Meiosis was induced in those cells, and the frequency of asci containing abnormal spores (<4 spores and/or premature spores with a thin spore wall) was measured 2 days later. At least 200 asci were examined for each strain; the mean values from three independent experiments are shown. Error bars represent the standard deviation. (**D**) Frequency of asci containing abnormal spores in *∆hta2* (TGO485) and *∆hta2* add-backed of *hta2*^+^ (TGO487; “*∆hta2* + *hta2*^+^”) cells. Meiosis was induced in those cells, and the frequency of asci containing abnormal spores was measured as in (**C**).

To examine the functional differences between H2Aα and H2Aβ, we constructed two strains with *hta1*^+^ and *hta2*^+^ deletions each (*∆hta1* and *∆hta2*). Cells of both *∆hta1* and *∆hta2* strains were viable and formed colonies comparable to those of wild type (WT) cells at 20 °C–36 °C (Fig. 1A). However, *∆hta2*, but not *∆hta1*, cells formed asci containing abnormal spores (<4 spores and/or premature spores with a thin spore wall) (Fig. 1B,C). Add-back of the *hta2*^+^ gene in *∆hta2* cells restored normal spore formation (Fig. 1D), confirming that the sporulation defect resulted from *hta2*^+^ deletion. These results indicated that *hta2*^+^ is specifically required for meiosis and sporulation.

**Figure 2:**
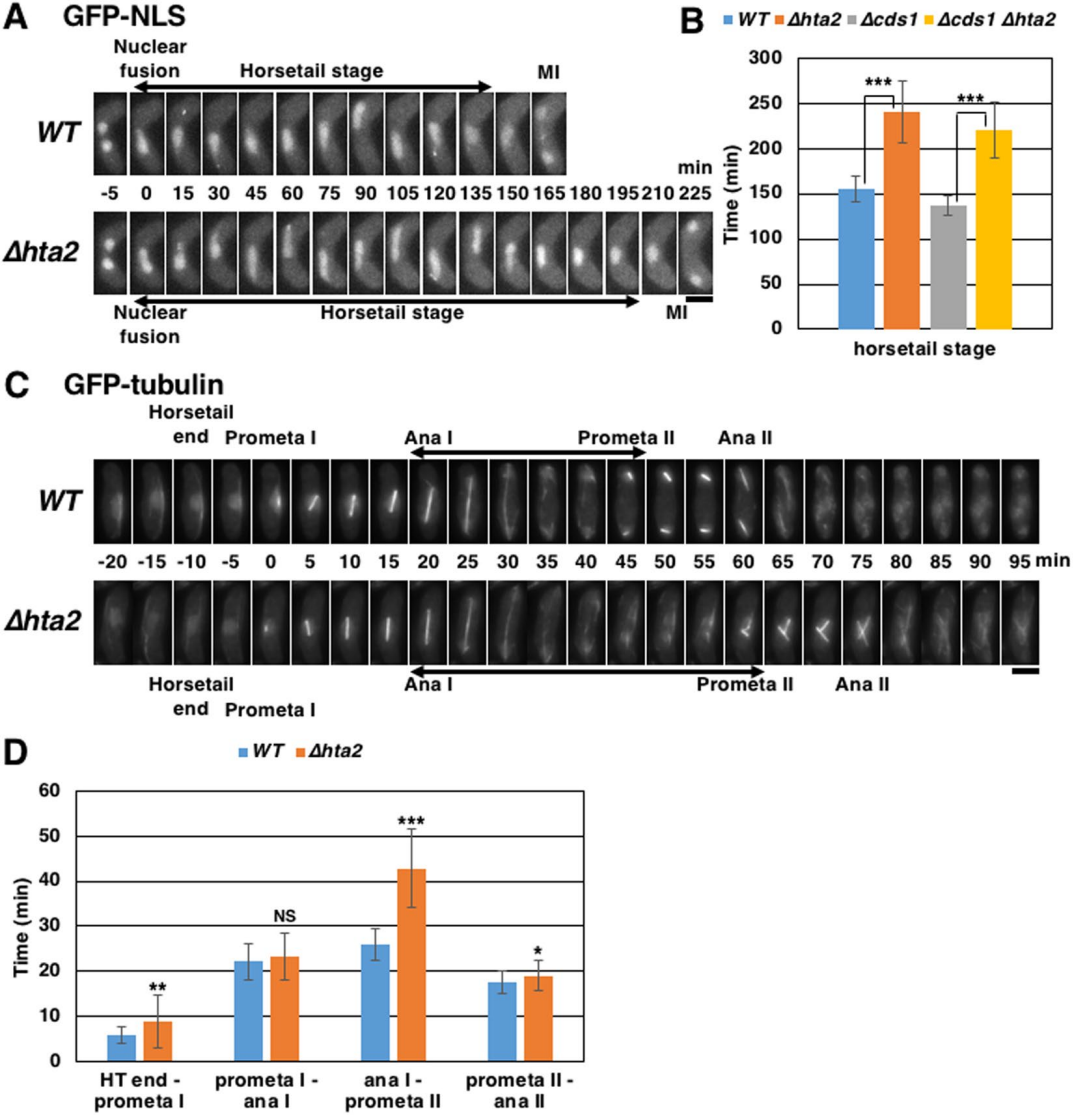
Prolonged meiosis in *∆hta2* cells. (**A**) Extended horsetail stage in *∆hta2* cells. Time-lapse images of meiosis progression from nuclear fusion to meiosis I in WT (TGO350) and *∆hta2* (TGO352) cells. The nucleus was labeled with GFP-NLS. Numbers indicate the time elapsed since nuclear fusion. MI indicates meiosis I timing. Scale bar, 5 µm. (**B**) Duration of the horsetail stage in WT (TGO350), *∆hta2* (TGO352), *∆cds1* (TGO542), and *∆cds1 ∆hta2* (TGO543) cells. At least 26 cells were examined for each strain; the mean values are shown. Error bars represent the standard deviation. (**C**) Extended period from anaphase I to prometaphase II in *∆hta2* cells. Time-lapse images of meiosis progression from the end of the horsetail stage to meiosis II in WT (TGO647) and *∆hta2* (TGO648) cells. Microtubules were labeled with GFP-tubulin. Numbers indicate the time elapsed since prometaphase I. “Prometa I”, “Ana I”, “Prometa II”, and “Ana II” indicate the timing of prometaphase I, anaphase I, prometaphase II, and anaphase II, respectively. Scale bar, 5 µm. (**D**) Durations from the end of the horsetail stage to prometaphase I (HT end–prometa I), prometaphase to anaphase I (prometa I–ana I), anaphase I to prometaphase II (ana I–prometa II), and prometaphase II to anaphase II (prometa II–ana II) in WT (TGO647) and *∆hta2* (TGO648) cells. At least 32 cells were examined for each strain; the mean values are shown. Error bars represent the standard deviation.

To examine the meiotic defects in *∆hta2* cells, we observed their nuclear behavior during meiosis progression in living *S. pombe* cells expressing green fluorescent protein fused to the nuclear localization signal (GFP-NLS) as a marker for the nucleus (Fig. 2). In *S. pombe* meiosis, fusion of two haploid nuclei was followed by the so-called “horsetail” nuclear movements (the elongated diploid nucleus moves back and forth within the cell), which in turn was followed by two consecutive nuclear divisions to form four spores, as shown for the WT example (Fig. 2A,C).

We first found extension of the horsetail stage in *∆hta2* cells (Fig. 2A). It was extended to 1.5 times in *∆hta2* cells than in WT cells (155 min in WT *vs*. 241 min in *∆hta2* cells) (Fig. 2B). Because the horsetail stage is reported to be extended upon DNA replication checkpoint activation^12^, we examined whether this extension in *∆hta2* cells was rescued by deleting the *cds1*^+^ gene for the DNA replication checkpoint. However, we still observed extension of the horsetail stage in *∆cds1 ∆hta2* cells (Fig. 2B), indicating that the DNA replication checkpoint is not involved in the extension of the horsetail stage observed in *∆hta2* cells.

We also found extension of the meiosis I–II duration in *∆hta2* cells. Therefore, we measured the meiosis I–II duration in cells expressing GFP-tubulin as a marker for the spindle to determine which meiotic period was extended. The prometaphase I-anaphase I duration was not extended in *∆hta2* cells (Fig. 2C,D), suggesting that the spindle assembly checkpoint is not activated. However, the anaphase I–prometaphase II duration was extended to 1.7 times in *∆hta2* cells than in WT cells (26 min in WT *vs*. 43 min in *∆hta2* cells) (Fig. 2C,D).

**Figure 3:**
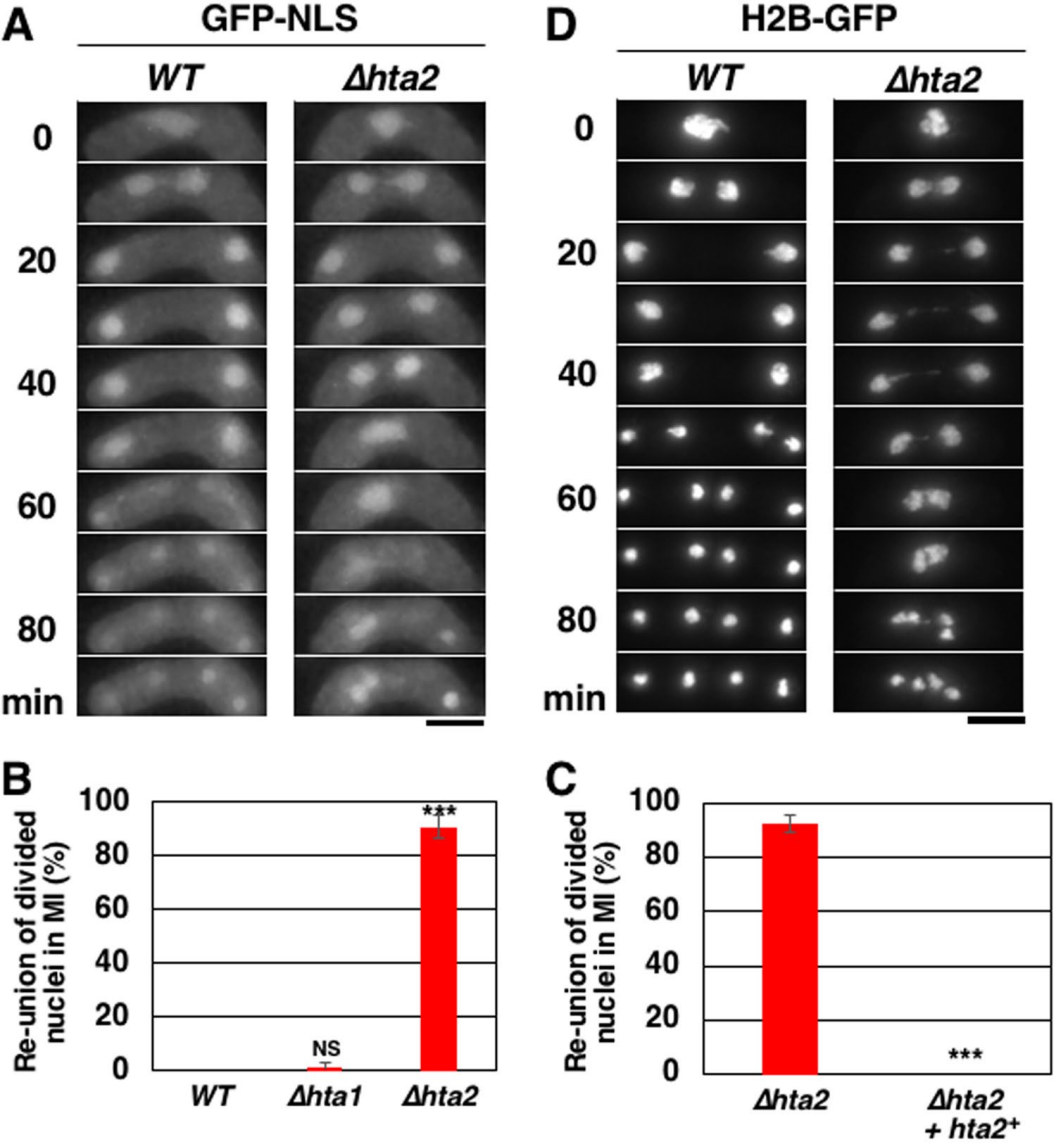
Abnormal nuclear division in meiosis I in *∆hta2* cells. (**A**) Time-lapse images of meiosis I progression in WT (TGO350) and *∆hta2* (TGO352) cells. The nucleus was labeled with GFP-NLS. Numbers indicate the time elapsed after anaphase I onset. Scale bar, 5 µm. (**B**) Frequency of cells showing reunion of divided nuclei in meiosis I in WT (TGO350), *∆hta1* (TGO351), and *∆hta2* (TGO352) cells. At least 20 cells were examined for each strain; the mean values from three independent experiments are shown. Error bars represent the standard deviation. (**C**) Frequency of cells showing reunion of divided nuclei in meiosis I in *∆hta2* (TGO485) and *∆hta2* add-backed of *hta2*^+^ (TGO487; “*∆hta2* + *hta2*”) cells. At least 30 cells were examined for each strain; the mean values from three independent experiments are shown. Error bars represent the standard deviation. (**D**) Time-lapse images of meiosis I progression in WT (TGO728) and *∆hta2* (TGO729) cells. The chromatin was labeled with H2B-GFP. Numbers indicate the time elapsed after anaphase I onset. Scale bar, 5 µm.

We observed the characteristic appearance of the spindles crossing over each other in the second division in *∆hta2* cells (Fig. 2C; 75 min in *∆hta2* cells). This characteristic spindle appearance was caused by abnormal nuclear division in meiosis I, in which the nucleus apparently divided into two daughter nuclei but reunited into a single nucleus in *∆hta2* cells (50 min in Fig. 3A). This reunion of divided nuclei in meiosis I was observed in most *∆hta2* cells but not in *∆hta1* cells (Fig. 3B). Add-back of the *hta2*^+^ gene in *∆hta2* cells resulted in the recovery of normal nuclear division in meiosis I (Fig. 3C), confirming that this nuclear division defect was due to the deletion of *hta2*^+^. These results indicated that *hta2*^+^ is required for normal meiosis progression and nuclear division in meiosis I.

Among the observed meiotic defects in *∆hta2* cells, we analyzed a failure in nuclear division (reunion of divided nuclei) that may be the major cause of the sporulation defect.

### *hta2*^+^ deletion causes chromosome bridge formation during nuclear division

To characterize the reunion of divided nuclei in *∆hta2* cells, we observed the behavior of chromosomes during meiotic nuclear divisions using GFP-tagged histone H2B (H2B-GFP), as shown in Fig. 3D. ∆*hta2* cells exhibited an anaphase chromosome bridge between two segregated masses of chromosomes (20–40 min in Fig. 3D, right). These chromosomes were reunited at the end of meiosis I (40–60 min in Fig. 3D, right) and segregated in meiosis II (80 min in Fig. 3D, right). This result suggested that entangled chromosomes in *∆hta2* cells resulted in the reunion of divided nuclei.

### Anaphase chromosome bridges form at rDNA repeats in ∆*hta2* cells

**Figure 4:**
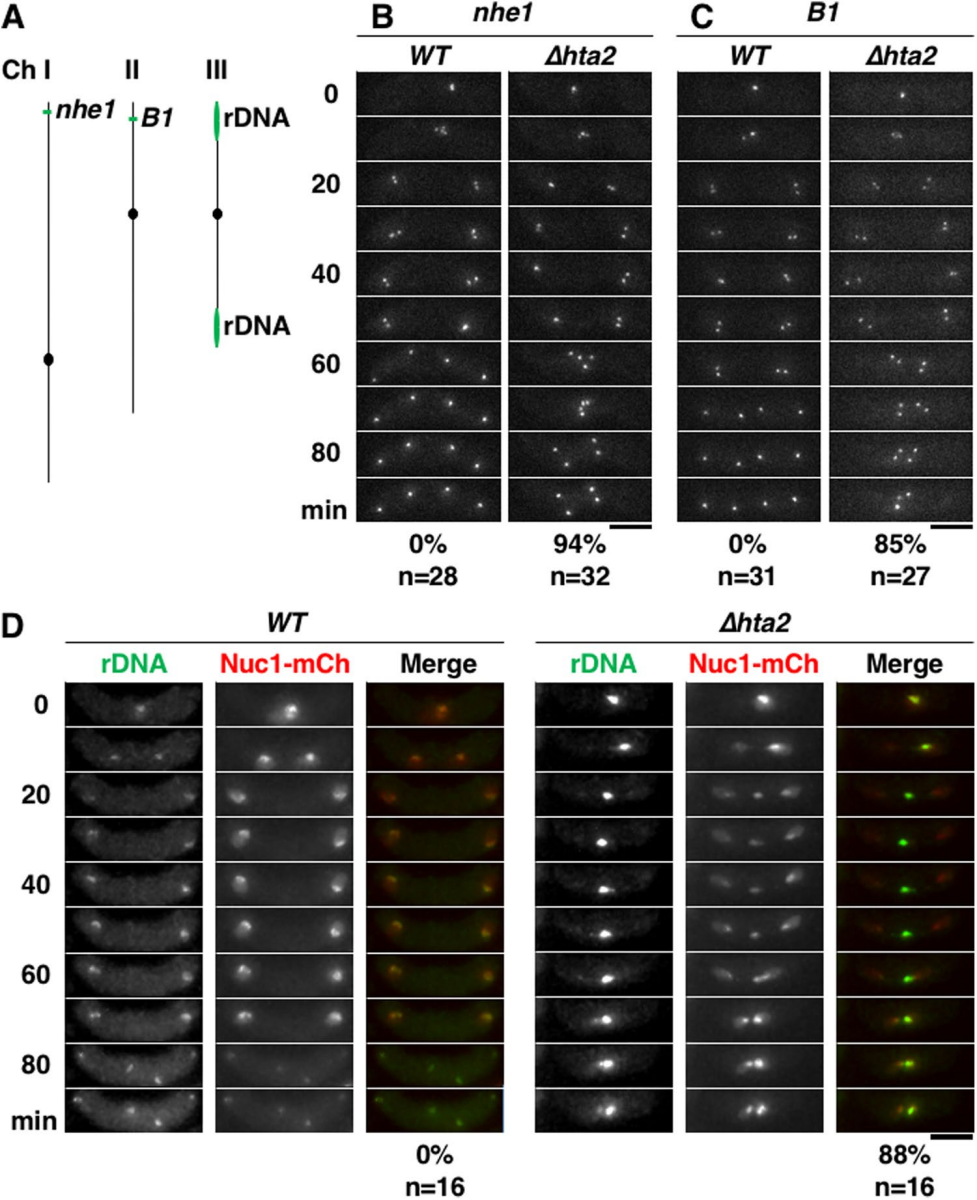
rDNA does not separate in meiosis I in *∆hta2* cells. (**A**) Schematic representing the positions of *nhe1*, *B1*, and rDNA loci on three chromosomes. The telomere–*nhe1* distance is ~51 kb, and the telomere–*B1* distance is ~110 kb. (**B**) Time-lapse images of meiosis I progression in WT (CT2121-4) and *∆hta2* (TGO462) cells. The *nhe1* locus was labeled with the LacI-GFP/*lacO* system. The numbers on the left of the images indicate the time elapsed after anaphase I onset, and the numbers under the images indicate the frequency of reunion of divided nuclei in meiosis I and total number of cells observed. Scale bar, 5 µm. (**C**) Time-lapse images of meiosis I progression in WT (YW537) and *∆hta2* (TGO463) cells. The *B1* locus was labeled with the LacI-GFP/*lacO* system. The numbers under the images indicate the frequency of reunion of divided nuclei in meiosis I and total number of cells observed. Scale bar, 5 µm. (**D**) Time-lapse images of meiosis I progression in WT (TGO566) and *∆hta2* (TGO578) cells. The rDNA and nucleolus were labeled with the LacI-GFP/*lacO* system (green, “rDNA”) and Nuc1-mCherry (red, “Nuc1-mCh”), respectively. The numbers on the left of the images indicate the time elapsed after anaphase I onset, and the numbers under the images indicate the frequency of reunion of divided nuclei in meiosis I and total number of cells observed. Scale bar, 5 µm.

As an anaphase chromosome bridge was observed, we assumed that chromosomes were entangled near the telomere. Therefore, we observed the behaviors of the *nhe1* (formerly called *sod2*) and *B1* (see the Methods section) loci near the telomeres of chromosomes I and II, respectively, that were visualized using the LacI-GFP/*lacO* system (Fig. 4A). Both loci segregated to the poles in *∆hta2* cells and were never observed within the anaphase chromosome bridge between the poles in 32 cells for *nhe1* and 27 cells for *B1* that we examined (Fig. 4B,C, right), indicating that the chromosomes were not entangled at the end of chromosome I or II. Nevertheless, the two divided nuclei reunited, suggesting that the chromosomes were entangled elsewhere.

Therefore, we next observed the behaviors of rDNA repeats located at both ends of chromosome III (Fig. 4A), visualized using the LacI-GFP/*lacO* system (see the Methods section). The rDNA repeats are surrounded by the nucleolus, and so we also visualized the nucleolus using mCherry tagged to a nucleolar protein Nuc1 (Nuc1-mCherry). The nucleus was also stained faintly by Nuc1-mCherry, allowing us to observe both the nucleus and nucleolus simultaneously. In WT cells, rDNA repeats and the nucleolus segregated normally during the two nuclear divisions (Fig. 4D, left). Conversely, an example of *∆hta2* cells (Fig. 4D, right) demonstrated that rDNA repeats and the nucleolus remained unsegregated in the middle of the cell, and a part of the nucleolus segregated into two nuclei (10–50 min in Fig. 4D, right); the segregated parts of the nucleolus moved towards one another and reunited at the center (60–80 min in Fig. 4D, right). These results indicated that anaphase chromosome bridges were formed at the rDNA repeats in *∆hta2* cells.

**Figure 5:**
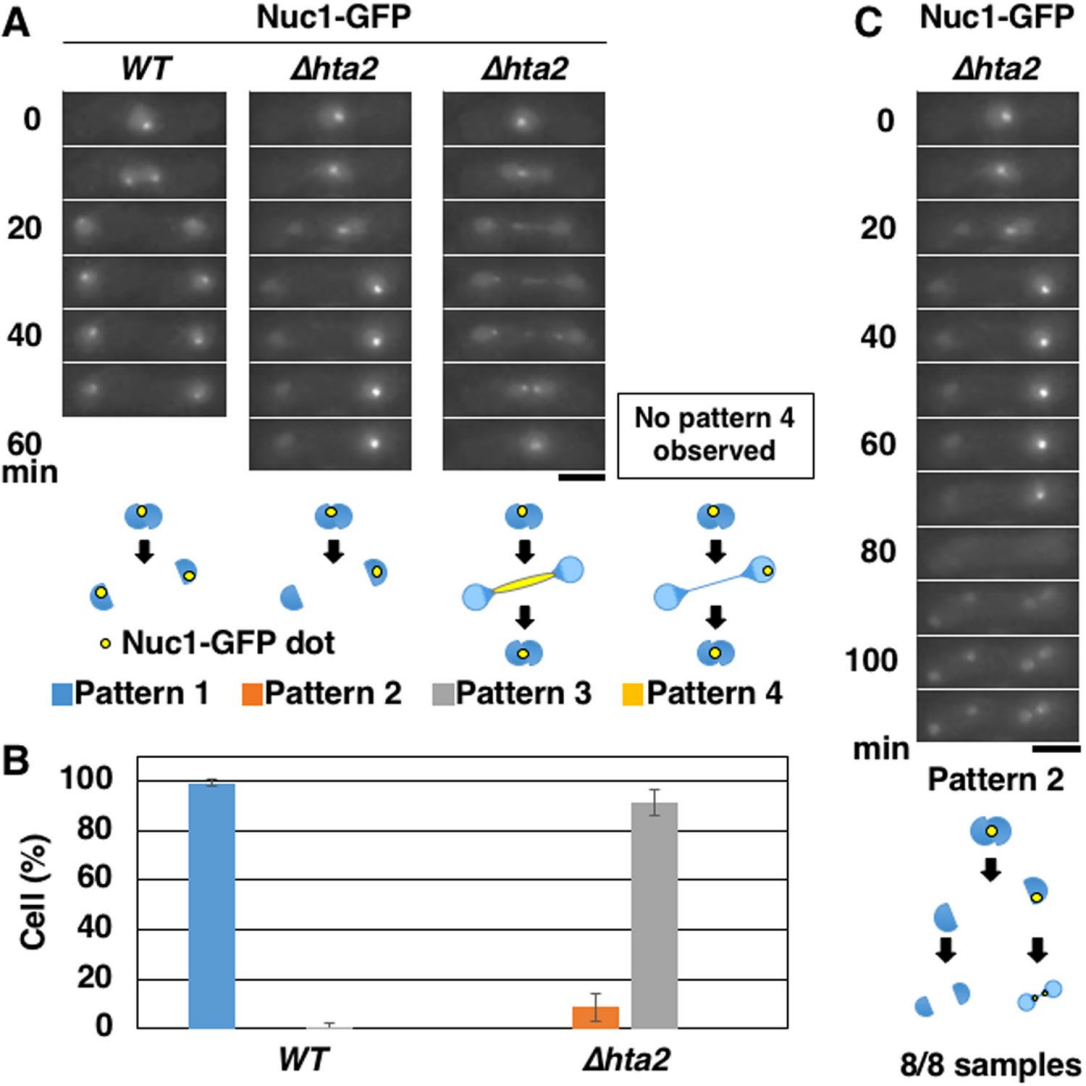
Characterization of the Nuc1 segregation pattern in *∆hta2* cells. (**A**) Time-lapse images of meiosis I progression in WT (TGO443) and *∆hta2* (TGO444) cells. The nucleolus was labeled with Nuc1-GFP. Patterns of segregation were classified into four categories based on nuclear division and Nuc1 segregation: normal nuclear division with divided nucleoli (pattern 1), nuclear division with the nucleolus remaining in one of the divided nuclei (pattern 2), reunion of divided nuclei with the connected nucleolus (pattern 3), and reunion of divided nuclei with the nucleolus remaining in one of the divided nuclei (pattern 4). Numbers indicate the time elapsed after anaphase I onset. Scale bar, 5 µm. (**B**) Frequency of cells showing the patterns 1–4 in meiosis I in WT (TGO443) and *∆hta2* (TGO444) cells. At least 28 cells were examined for each strain; the mean values from three independent experiments are shown. Error bars represent the standard deviation. (**C**) Time-lapse images of meiosis I and II progression in *∆hta2* pattern 2 cells shown in (**A**). The nucleolus was labeled with Nuc1-GFP. Numbers indicate the time elapsed after anaphase I onset. Scale bar, 5 µm.

Although we observed the phenotype of the reunion of divided nuclei in the majority of *∆hta2* cells, we also observed the formation of two daughter nuclei in a small part of the cells. Therefore, we counted the populations of these classes of phenotypes in Nuc1-GFP-expressing cells. We expected four patterns of the phenotypes shown in Fig. 5A and categorized the images into the following four patterns: normal nuclear division with divided nucleoli (pattern 1), nuclear division with the nucleolus remaining in one of the divided nuclei (pattern 2), reunion of divided nuclei with the connected nucleolus (pattern 3), and reunion of divided nuclei with the nucleolus remaining in one of the divided nuclei (pattern 4). In WT cells, we observed normal segregation of Nuc1-GFP (pattern 1) in 104 out of 105 cells examined (Fig. 5B). In contrast, the majority of *∆hta2* cells (91%) showed the reunion of divided nuclei with the connected nucleolus (pattern 3); in some cells (9%), the nucleus divided into two, and the nucleolus was observed only in one of the divided nuclei (pattern 2) (Fig. 5B). In pattern 2 cells, nondisjunction of chromosome III must occur, suppressing the pattern 3 phenotype. We did not observe any examples of the reunion of divided nuclei with the nucleolus in one of the nuclei (pattern 4). These findings indicated that all *∆hta2* cells observed showed defects in meiosis I (pattern 2 or 3).

We observed that in the second meiotic division of pattern 2 *∆hta2* cells (Fig. 5C), the nucleus containing the nucleolus failed to divide (the right nucleus), while the nucleus without the nucleolus divided into two nuclei (the left nucleus). This behavior of nuclear division in meiosis II was observed in all pattern 2 cells examined (8 in eight cases). These findings supported our idea that the anaphase chromosome bridge formed at rDNA repeats led to the reunion of divided nuclei.

### Defects in ∆*hta2* cells are rescued by increased *hta1* expression

**Figure 6:**
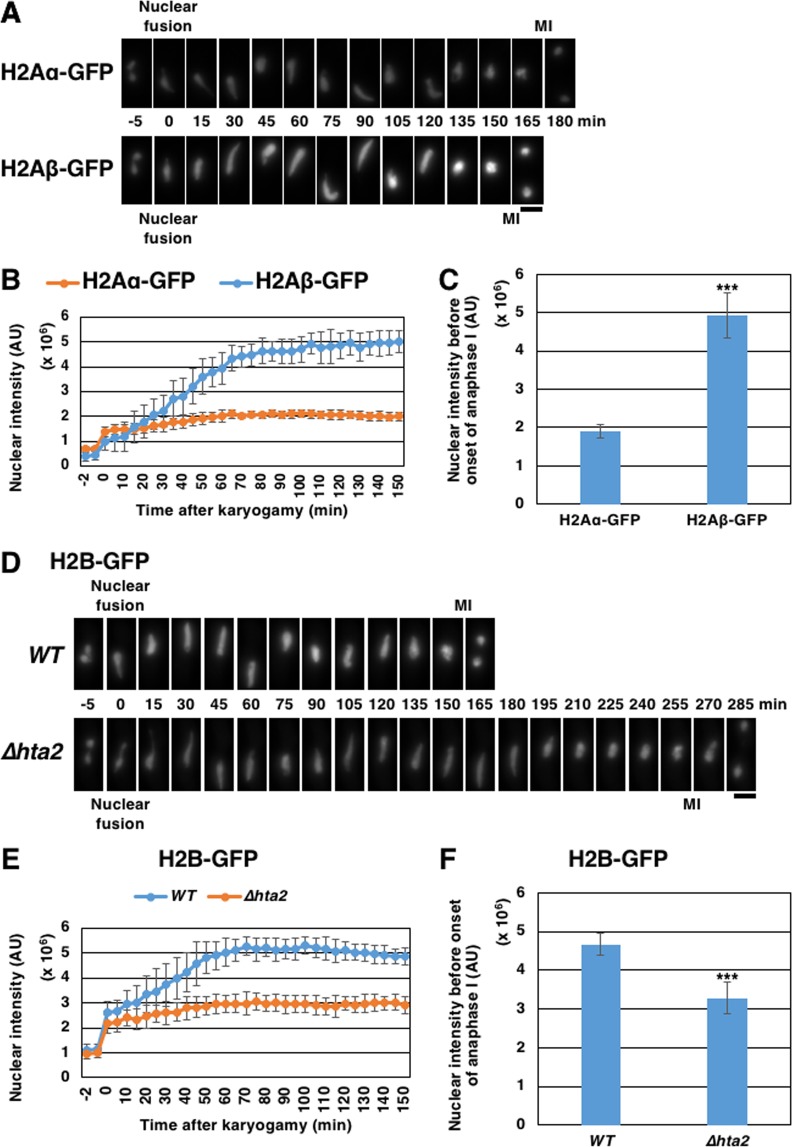
Amounts of histone H2Aβ increase in meiosis. (**A**) Time-lapse images of meiosis progression from nuclear fusion to meiosis I in WT cells expressing H2Aα-GFP (TGO804) or H2Aβ-GFP (TGO808). Numbers indicate the time elapsed since nuclear fusion. MI indicates meiosis I timing. Scale bar, 5 µm. (**B**) Time course of the nuclear intensity of H2Aα-GFP (TGO804) and H2Aβ-GFP (TGO808). (**C**) Nuclear intensity of H2Aα-GFP (TGO804) or H2Aβ-GFP (TGO808) before anaphase I onset in WT cells. For (**B**) and (**C**), at least nine cells were examined for each strain; the mean values are shown. Error bars represent the standard deviation. (**D**) Time-lapse images of meiosis progression from nuclear fusion to meiosis I in WT (TGO728) and *∆hta2* (TGO729) cells expressing H2B-GFP. Numbers indicate the time elapsed since nuclear fusion. MI indicates meiosis I timing. Scale bar, 5 µm. (**E**) Time course of the nuclear intensity of H2B-GFP in WT (TGO728) and *∆hta2* (TGO729) cells. (**F**) Nuclear intensity of H2B-GFP before anaphase I onset in WT (TGO728) and *∆hta2* (TGO729) cells. For (**E** and **F**), at least 15 cells were examined for each strain; the mean values are shown. Error bars represent the standard deviation.

As the mRNA levels of *hta2*^+^, but not of *hta1*^+^, are upregulated in meiosis^11^, we estimated the H2Aα and H2Aβ levels by measuring the fluorescence intensity of the GFP-fused proteins (H2Aα-GFP and H2Aβ-GFP, respectively) during meiosis. We found that the fluorescence intensity of H2Aβ-GFP in the nucleus increases more strikingly compared to that of H2Aα-GFP during meiosis progression (Fig. 6A,B). We also compared the fluorescence intensities of H2Aα-GFP and H2Aβ-GFP in the nucleus at anaphase I onset. We found that the fluorescence intensity of H2Aβ-GFP was 2.6-fold higher than that of H2Aα-GFP (Fig. 6C). These results were consistent with those of a previous transcriptional study^11^. Similarly, we measured the fluorescence intensity of H2B-GFP and found that the fluorescence intensity of H2B-GFP in the nucleus also increased during meiosis but remained low in *∆hta2* cells (Fig. 6D–F). These results suggested that the histone H2A and H2B levels in the nucleus increase during meiosis and reduce in the absence of *hta2*^+^. Therefore, it is possible that histone H2A levels are insufficient in *∆hta2* cells during meiosis and that reduced histone H2A levels result in the reduction of histone H2B, possibly leading to insufficiency of the H2A–H2B dimer.

**Figure 7:**
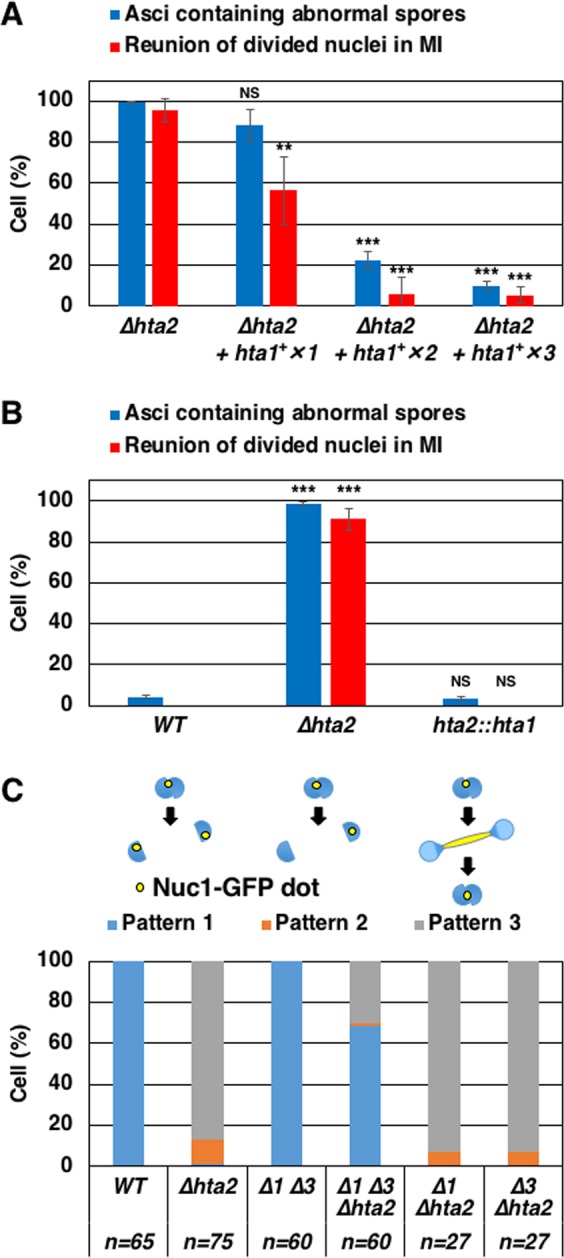
Additional expression of *hta1*^+^ rescues defects in *∆hta2* cells. (**A**) Frequency of asci containing abnormal spores and reunion of divided nuclei in meiosis I in *∆hta2* cells expressing no extra copy (TGO629; “*∆hta2*”), one extra copy (TGO630; “*∆hta2* + *hta1*^+^ ×*1*”), two extra copies (TGO631; “*∆hta2* + *hta1*^+^ ×*2*”), or three extra copies (TGO632; “*∆hta2* + *hta1*^+^ ×*3*”) of *hta1*^+^. Abnormal spore formation and nuclear division in *∆hta2* cells were suppressed by expression of additional copies of *hta1*^+^. At least 200 asci or 31 cells were examined for spore formation or nuclear division, respectively, for each strain; the mean values from three independent experiments are shown. Error bars represent the standard deviation. (**B**) Frequency of asci containing abnormal spores and reunion of divided nuclei in meiosis I in WT (TGO350), *∆hta2* (TGO352), and *hta2::hta1* (TGO399) cells. Abnormal spore formation and nuclear division were not observed in cells that replaced the *hta2* ORF with the *hta1* ORF (“*hta2::hta1”*). At least 200 asci or 29 cells were examined for spore formation or nuclear division, respectively, for each strain; the mean values from three independent experiments are shown. Error bars represent the standard deviation. (**C**) Frequency of cells showing patterns 1–3 in meiosis I in WT (TGO477), *∆hta2* (TGO521), *∆1 ∆3* (TGO522), *∆1 ∆3 ∆hta2* (TGO523), *∆1 ∆hta2* (TGO525), or *∆3 ∆hta2* (TGO526) cells. rDNA was labeled with Nuc1-GFP. Patterns of segregation were classified into four categories based on nuclear division and Nuc1 segregation, as in Fig. 5A. The number of cells examined is shown at the bottom of the graph.

To investigate whether histone H2A insufficiency is a cause of meiotic defects in *∆hta2* cells, we constructed *∆hta2* strains bearing additional copies of *hta1*^+^. The strain bearing one additional copy of *hta1*^+^ showed slightly decreased abnormal spore formation and nuclear division (“*hta1*^+^ ×*1*” in Fig. 7A). Strikingly, increasing the copies of *hta1*^+^ more remarkably eliminated these defects (“*hta1*^+^ ×*2*” and “*hta1*^+^ ×*3*” in Fig. 7B). We also examined this by expressing *hta1*^+^ under the *hta2* promoter by replacing the *hta2* coding region with the *hta1* coding region (see the Methods section; “*hta2::hta1*” in Fig. 7B). We observed that abnormal spore formation and nuclear division in *∆hta2* cells was eliminated in *hta2::hta1* cells (Fig. 7B). These results indicated that the phenotypes uniquely observed in *∆hta2* cells were not due to differences in amino acid residues between H2Aα and H2Aβ but due to histone H2A insufficiency.

### Reduction of histone H3 and H4 partially rescues the chromosome segregation errors in ∆*hta2* cells

To examine involvement of histone H3 and H4 in ∆*hta2* cells, we deleted 1 or 2 of the 3 genes for histone H3 and H4. Among the three pairs of histone H3 and H4 genes, we deleted a pair of the *hhf1*^+^ and *hht1*^+^ genes (*∆1*) and/or a pair of the *hhf3*^+^ and *hht3*^+^ genes (*∆3*). Cells of *∆1* ∆*hta2* and *∆3* ∆*hta2* showed a high frequency of abnormal nuclear division (pattern 2 and 3) at a level similar to that in ∆*hta2* cells; however, this defect was partially rescued in *∆1 ∆3* ∆*hta2* cells (Fig. 7C), suggesting that depletion of histone H3 and H4 rescues histone H2A insufficiency to a limited extent. Thus, excess amounts of histone H3 and H4 as a consequence of H2A and H2B insufficiency may be a cause of chromosome segregation defects.

### Histone H2A insufficiency also causes chromosome bridge formation at rDNA repeats during mitosis

**Figure 8:**
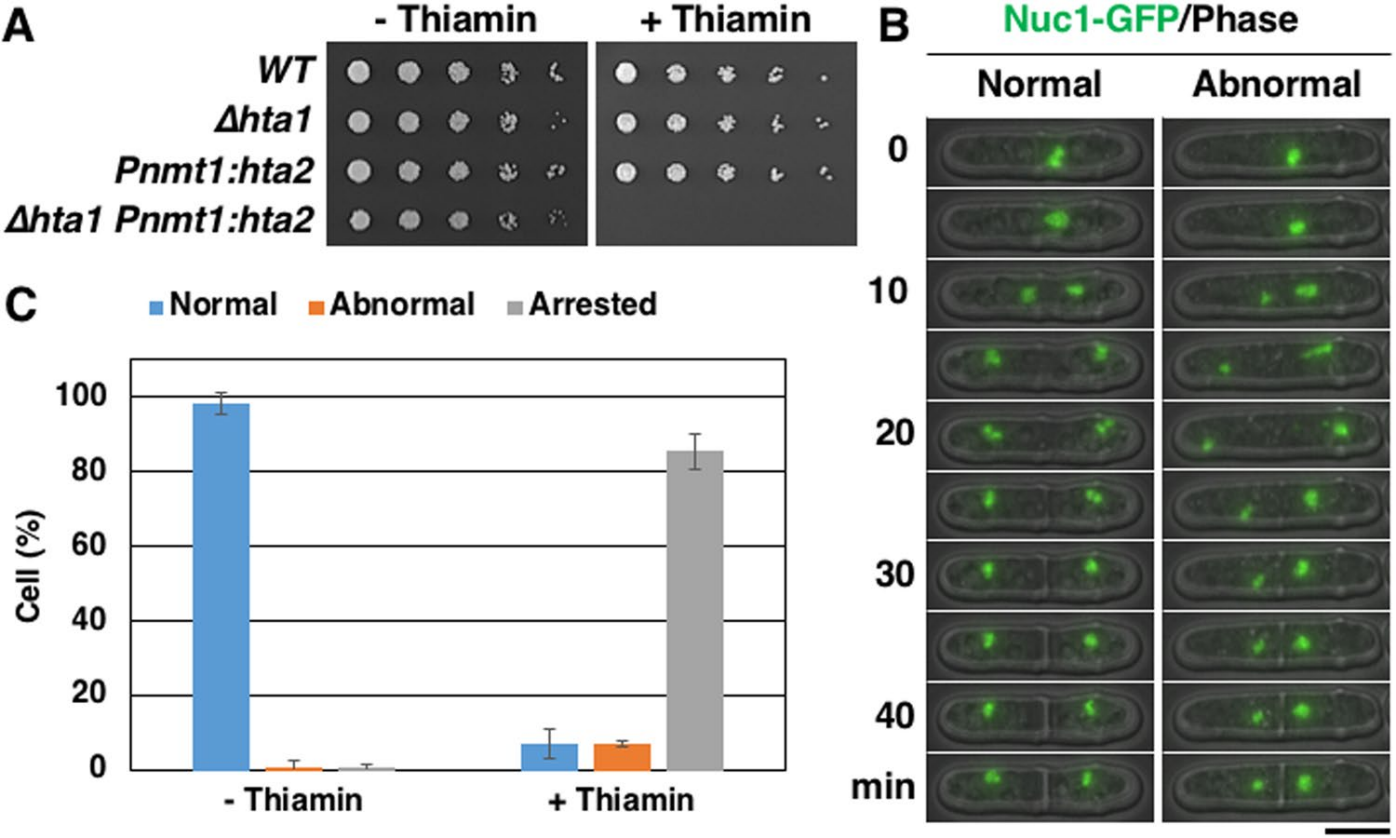
Depletion of histone H2A in mitosis. (**A**) Spot assay comparing the growth of WT (TGO443), *∆hta1* (TGO575), *Pnmt1:hta2* (TGO572), and *∆hta1 Pnmt1:hta2* (TGO579) cells. Dilution series (1/5 dilution) of cell suspensions were spotted on EMM2 with (“+Thiamin”) or without (“−Thiamin”) thiamin and grown for 3 days at 30 °C. (**B**) Time-lapse images of progression of normal and abnormal mitosis in *∆hta1 Pnmt1:hta2* (TGO579) cells. The nucleolus was labeled with Nuc1-GFP. Numbers indicate the time elapsed after anaphase onset. Scale bar, 5 µm. (**C**) Frequency of normal and abnormal nuclear divisions at the second mitosis during observation in *∆hta1 Pnmt1:hta2* (TGO579); frequency of arrested cells (no second mitosis during a 12 h observation) is also shown. At least 72 cells were examined for each condition; the mean values from three independent experiments are shown. Error bars represent the standard deviation.

Finally, we examined whether histone H2A insufficiency causes chromosome bridge formation at rDNA repeats during mitosis. We constructed a strain in which *hta2*^+^ expression could be conditionally shut down using the *nmt1* promoter under the *∆hta1* background (i.e., *hta2* expression could be induced by the absence of thiamin and be repressed by the addition of thiamin). As expected, this strain did not form a colony on the plate containing thiamin (Fig. 8A). Because ~80% of the cells were in the G2 phase in the *S. pombe* asynchronous culture, most cells had sufficient histone H2A to perform one nuclear division. Therefore, we focused on the second nuclear division after the addition of thiamin to examine the cells depleted of H2A. Although most cells proceeded with a normal second nuclear division in the absence of thiamin (Fig. 8B,C), they did not enter the second nuclear division in the presence of thiamin (Fig. 8C). In some of the cells that did enter the second nuclear division, the nuclear division failed as observed by Nuc1-GFP (Fig. 8B, “Abnormal”). In these cells, the nucleus apparently divided into two daughter nuclei (10–20 min in Fig. 8B, “Abnormal”) but returned to the center of the cells (25 min in Fig. 8B, “Abnormal”), as observed in *∆hta2* cells, although the reunion of divided nuclei was disturbed by septation (30 min in Fig. 8B, “Abnormal”). These results indicate that histone H2A insufficiency causes chromosome segregation errors at rDNA repeats during mitosis as well, suggesting that this phenotype is not meiosis-specific.

## Discussion

In this study, we demonstrated that deletion of *hta2*^+^ causes meiotic defects but no remarkable defects in vegetative growth. This does not indicate that histone H2Aβ produced from *hta2*^+^ has meiosis-specific functions; instead, the defects occur due to histone H2A insufficiency. This finding is supported by the fact that when increased amounts of H2Aα is produced by *hta1*^+^, it can replace histone H2Aβ. Therefore, to date, no functional differences between H2Aα and H2Aβ have been found.

We first considered the involvement of condensin in chromosome segregation defects in *∆hta2* cells. Studies have reported that histone H2A binds to condensin^10^; therefore, histone H2A insufficiency might cause insufficient loading of condensin on to chromatin, leading to chromosome segregation defects. However, we concluded that condensin is not involved because a mutant of histone H2Aβ (K13A R18A K21A) that does not bind to condensin^10^ rescued the defects in *∆hta2* cells (Supplementary Fig. S1). Similarly, a mutant of histone H2Aβ (S121A) that does not recruit shugoshin^9^ rescued the defects in *∆hta2* cells (Supplementary Fig. S1). In addition, a mutant of H2Aβ (S127A) that lacks the phosphorylation site required for DNA damage repair^8^ also rescued the defects in *∆hta2* cells (Supplementary Fig. S1), indicating that condensin loading was not involved in chromosome segregation defects in *∆hta2* cells.

We then considered the involvement of cohesin in chromosome segregation defects in *∆hta2* cells. In meiosis, mitotic cohesin Rad21 is replaced to a large extent with meiotic cohesin Rec8^13^; however, Rad21 remains at the chromosomal regions of rDNA repeats during the horsetail stage^14^. The frequency of Rad21 localization to rDNA repeat regions was increased in ∆*hta2* cells (Supplementary Fig. S2). This increased localization of Rad21 could be a cause for the observed defects. However, the frequency of abnormal nuclear division in *∆rad21 ∆hta2* cells was high and at a level similar to that in *∆hta2* (Supplementary Fig. S2), indicating that increased localization of Rad21 at the rDNA repeat regions was not a cause of chromosome segregation defects in *∆hta2*.

The characteristic phenotype of the reunion of divided nuclei during meiosis I as observed in *∆hta2* cells has also been reported in the *∆dbl2* mutant^15^. However, unlike that in the *∆hta2* mutant, rDNA repeats were not involved in the *∆dbl2* mutant as observed by Nuc1-GFP (Supplementary Fig. S3), indicating that the role of histone H2A is unrelated to the Dbl2 pathway, which regulates the resolution of recombination intermediates during meiosis^15^.

Alternatively, transcription of rRNA genes might cause chromosome segregation defects at rDNA repeats. Chromosome regions at rDNA repeats have sparse nucleosomes, which occupy only the intervening sequences between 18S and 28S rRNA coding sequences^16^. Upon entry into meiosis, rRNA transcription might be repressed by the occupation of nucleosomes on rRNA genes. Anaphase chromosome bridge formation at rDNA repeats was also observed in a *cdc14* mutant in *Saccharomyces cerevisiae*^17,18^. Cdc14 is a mitotic phosphatase^19^ and inhibits rRNA transcription by polymerase I to separate the rDNA repeats^20^. Inhibition of rRNA transcription by thiolutin rescues chromosome bridge formation at rDNA repeats in a *cdc14* mutant in *S. cerevisiae*^21^. Therefore, histone H2A insufficiency might lead to failure of transcriptional repression for rRNA genes and consequently cause chromosome segregation failure at rDNA repeats in *S. pombe*. However, thiolutin treatment did not suppress chromosome bridge formation at rDNA repeats in *∆hta2* cells (Supplementary Fig. S4), suggesting that transcription of rDNA repeats is not the cause of chromosome bridge formation in *S. pombe*.

Histone stoichiometry is considered important for the fidelity of chromosome segregation in *S. cerevisiae*^22^. In *S. cerevisiae*, canonical histone proteins are encoded by multiple genes. The increased copy number of histone genes results in chromosome loss^22^, while a decrease in the copy number of the genes causes slow growth and G2/M arrest^23–25^. The dosage of histone proteins affects the cell sensitivity to DNA damaging agents: an excess amount of histones enhances sensitivity, whereas a reduced amount increases resistance^26^ and histone H4 depletion induces hyper-recombination, collapse of replication forks, and activation of the spindle assembly checkpoint, leading to genome instability^27–29^. Thus, it is likely that histone imbalance is responsible for the defects associated with chromosome instability in *S. pombe* as well.

Considering that chromosome regions at rDNA repeats have sparse nucleosomes^16^, these regions may be sensitive to histone imbalance. Measurements of nucleosome occupancy at rDNA repeats in strains bearing histone imbalance may reveal the cause of chromosome segregation errors associated with histone imbalance.

## Methods

### Strains and culture media

The *S*. *pombe* strains used in this study are listed in Supplementary Table S1. The growth media and basic genetic techniques for *S*. *pombe* have been described in previous studies^30^. The complete yeast extract with supplements (YES) medium (i.e., YE medium supplemented with 225 mg/L of adenine, leucine, histidine, uracil, and lysine) was used for spot assay (Fig. 1A). Edinburgh Minimal Media (EMM2) containing nutritional supplements (150 mg/L of adenine, 200 mg/L of leucine, and 75 mg/L of lysine), when necessary, was used for routine culture. EMM2 lacking nitrogen sources (EMM2-N) containing nutritional supplements (150 mg/L of adenine, 200 mg/L of leucine, and 75 mg/L of lysine), when necessary, was used to induce meiosis. The frequency of abnormal nuclear division at the first meiosis in *∆hta2* cells is high (~90%) if meiosis is induced on an EMM2-N plate but moderate (40–60%) on a molt extract plate. EMM2 was supplemented with 2 µM thiamin to repress the *nmt1* promoter and depleted of thiamin to induce the *nmt1* promoter^31^.

### Strain constructions

*S. pombe* strain expressing GFP-NLS (NLS from the SV40 T-antigen) was constructed as follows: First, the *nmt1* promoter and the GFP coding region of pCST8^32^ were replaced with the *nda3* promoter (−620 to −1 nucleotides [nt]) and the GFP-NLS coding region^33^, respectively. Next, the resulting plasmid, pTG3, was integrated into the chromosome at the *lys1* locus. Integration was confirmed by polymerase chain reaction (PCR).

*S. pombe* strains expressing *hta1* from the *lys1*, *leu1*, and *aur1* loci were constructed as follows: First, the *hta1* gene fragment containing its promoter (from −626 nt), coding region, and terminator (601 nt after the stop codon) was ligated into the integration vectors pYC36^34^, pYC28^33^, and pYC33; pYC33 is a derivative of pYC36 and has the *aur1R* gene fragment (TaKaRa Bio Inc., Shiga Prefecture, Japan) containing its partial coding region (72 nt to the stop codon) and terminator (639 nt after the stop codon) instead of the *lys1-N* fragment. Next, the resulting plasmids, namely, pYC36-hta1, pYC28-hta1, and pYC33-hta1, were integrated into the chromosome at the *lys1*, *leu1*, and *aur1* loci, respectively. Integration was confirmed by PCR.

*S. pombe* strain expressing *hta2* from the *lys1* locus was constructed as follows: First, the *hta2* gene fragment containing its promoter (from −561 nt), coding region, and terminator (603 nt after the stop codon) was ligated into the integration vector pYC36. Next, the resulting plasmid, pYC36-hta2, was integrated into the chromosome at the *lys1* locus. Integration was confirmed by PCR.

*S. pombe* strain expressing *htb1* from the *lys1* locus was constructed as follows: First, the *htb1* gene fragment containing its promoter (from −471 nt), coding region, and terminator (693 nt after the stop codon) was ligated into the integration vector pYC36. Next, the resulting plasmid, pYC36-htb1, was integrated into the chromosome at the *lys1* locus. Integration was confirmed by PCR.

*S. pombe* strains deleted of *hta1* and *hta2* gene (*∆hta1* and *∆hta2*, respectively) were constructed using PCR-based gene targeting^35,36^. Gene deletion was confirmed by PCR and sequencing.

*S. pombe* strain expressing *hta1* under the *hta2* promoter (*hta2::hta1*) was constructed as follows: First, the *hta2* coding region of pYC36-hta2 was replaced with the *hta1* coding region. Next, the *hta2::hta1* fragment from the resulting plasmid, pYC36-P2-hta1-T2, was amplified by PCR and transformed to an *hta2* gene deletion strain (*∆hta2::ura4*). Finally, transformants were selected by 5-fluoroorotic acid (5-FOA) and confirmed by sequencing.

*S. pombe* strain expressing *hta2* under the *nmt1* promoter (*Pnmt1:hta2*) was constructed as follows: First, the *nmt1* promoter of pCST8 was inserted in the *Sac*I site of pAG32^37^. Next, the resulting plasmid, pAG32-Pnmt1, was used for PCR-based gene targeting to integrate the *nmt1* promoter fragment at the *hta2* locus. Integration was confirmed by PCR.

*S. pombe* strain expressing H2Aα-GFP was constructed as follows: First, the *hta1-GFP* fragment containing the *hta1* promoter (from −626 nt), *hta1* and *GFP* coding regions, and the *hta1* terminator (601 nt after the stop codon) was cloned into plasmid pHSG299 using the In-Fusion HD Cloning Kit. Next, the *hta1-GFP* fragment from the resulting plasmid, pHSG-hta1-GFP, was amplified by PCR and transformed to an *hta1* gene deletion strain (*∆hta1::ura4*). Finally, transformants were selected by 5-FOA and confirmed by sequencing.

*S. pombe* strain expressing H2Aβ-GFP was constructed as follows: First, the *hta2-GFP* fragment containing the *hta2* promoter (from −561 nt), *hta2* and *GFP* coding regions, and the *hta2* terminator (603 nt after the stop codon) was cloned into plasmid pHSG299 using the In-Fusion HD Cloning Kit. Next, the *hta2-GFP* fragment from the resulting plasmid, pHSG-hta2-GFP, was amplified by PCR and transformed to an *hta2* gene deletion strain (*∆hta2::ura4*). Finally, transformants were selected by 5-FOA and confirmed by sequencing.

*S. pombe* strain expressing H2B-GFP was constructed as follows: First, the *htb1-GFP* fragment from the *htb1-GFP* plasmid^38^ was amplified by PCR and then transformed to an *htb1* gene deletion strain (*∆htb1::ura4*) integrated pYC36-htb1. Then, transformants were selected by 5-FOA and confirmed by sequencing.

In this study, chromosomal loci were visualized using the lac repressor (LacI)/lac operator (*lacO*) recognition system^39–41^. To track the dynamics of the telomere-proximal locus of chromosome II, tandem repeats of *lacO* arrays were integrated in the position of chromosome II between 110,029 and 110,061 using two-step integration^42^. This locus was named *B1*, and integration was confirmed by PCR.

To track the dynamics of rDNA in live cells, 5x *lacO* arrays were integrated into the repetitive 28s ribosomal RNA (rRNA) gene cluster using the I-PpoI cut-and-refill method^43,44^ and visualized with LacI-GFP. I-PpoI is a homing endonuclease encoded by the group I intron^45^. In *S. pombe*, in addition to other eukaryotes, the 28s rRNA gene contains a unique 15 bp target sequence of I-PpoI. Induction of I-PpoI makes double-strand breaks at rDNA repeats and kills the cell unless the target site is repaired and mutated. Next, a 28s rRNA gene and TsLSU-5x *lacO* containing a DNA fragment, in which the I-PpoI recognition site was interrupted by TsLSU-5x *lacO* insertion (cloned from plasmid p5xlacOTtLSU^43^, a gift from Dr. Yu), were cloned into plasmid pREP41^46^ using the In-Fusion HD Cloning Kit, and the resulting plasmid was named pY12. pY12 was transformed to LacI-GFP-containing cells and selected on a leucine dropout plate. The resulting cells were then transformed with pSS12^44^, a tetracycline-inducible I-PpoI expression plasmid (a gift from Dr. Sanders). The I-PpoI-resistant cell, in which the I-PpoI recognition site was disrupted and in which 5x *lacO* was inserted, was selected on an ahTET-containing plate^44^ and confirmed with colony PCR and sequencing. GFP fluorescence at rDNA was confirmed using a microscope. The cells were cultured in a rich medium for at least 20 generations to drop out plasmids pY12 and pSS12, which were subsequently selected on a 5-FOA plate.

### Live-cell fluorescence microscopy

Microscopy images were obtained using a DeltaVision microscope system (GE Healthcare, Chicago, IL, USA) equipped with a Plan Apo 60x oil-immersion objective lens (numerical aperture [NA] = 1.4; Olympus Corporation, Tokyo, Japan) and a CoolSNAP HQ2 CCD (Photometrics, Tucson, AZ, USA). For time-lapse observation, we mounted living cells on 35 mm glass-bottomed culture dishes (MatTek Corporation, Ashland, MA, USA) coated with 0.2 mg/mL of soybean lectin (Sigma-Aldrich Corporation, St. Louis, MO, USA) and observed them at 26 °C. Briefly, we took a set of images of 11 focal planes at 0.3 µm intervals every 5 min. To measure the meiosis duration, we took a set of images of seven focal planes at 0.5 µm intervals every 5 min. To quantify H2A-GFP and H2B-GFP, we took a set of images of 11 focal planes at 0.4 µm intervals every 5 min and measured nuclear fluorescence intensities, as described previously^47^, with the following threshold values to draw two-dimensional (2D) polygons: 1800 for H2Aα-GFP, 3800 for H2Aβ-GFP, and 3500 for H2B-GFP. We used a semiconductor light source instead of a mercury arc; therefore, we detected almost no progressive decline of light output during our observation for quantification. All images, except those in Figs 1B, 3D and 6A,D, were processed using the denoising algorithm^48^ and then projected with a maximum intensity method. The images in Fig. 1B were projected with a maximum intensity method without denoising, and those in Figs 3D and 6A,D were projected with a summation method without denoising. Projection and quantification were performed using softWoRx software (GE Healthcare).

### Statistical analyses

All statistical analyses were performed using R (www.r-project.org). For between-group comparison, we used two-sided, unpaired Student’s *t*-test. For multiple-group comparison, we used two-sided Tukey’s (Tukey–Kramer). Supplementary Table S2 summarizes the types of test methods and *P*-values in each analysis. A significance level (*α*) was set at 0.05 in all analyses, and significance was indicated by asterisks in all graphs: *P < 0.05, **P < 0.01, and ***P < 0.001. NS stands for “not significant” (*P* ≥ 0.05).

## Supplementary information

Supplementary Information
