# Supplementary material for: Histone H2A insufficiency causes chromosomal segregation defects due to anaphase chromosome bridge formation at rDNA repeats in fission yeast
Source: Sci Rep. 2019 May 9;9:7159. doi: 10.1038/s41598-019-43633-5 (PMC6509349; doi:10.1038/s41598-019-43633-5)
Supplement: Supplementary file 1 — Supplementary Information [file 41598_2019_43633_MOESM1_ESM.pdf]

Supplementary Information

Histone H2A insufficiency causes chromosomal segregation defects due to anaphase chromosome bridge formation at rDNA repeats in fission yeast

Takaharu G. Yamamoto[1], Da-Qiao Ding[1], Yuki Nagahama[1], Yuji Chikashige[1], Tokuko Haraguchi[1,2] and Yasushi Hiraoka[1,2]

[1] Advanced ICT Research Institute Kobe, National Institute of Information and Communications Technology, 588-2 Iwaoka, Iwaoka-cho, Nishi-ku, Kobe 651-2492, Japan

[2] Graduate School of Frontier Biosciences, Osaka University, 1-3 Yamadaoka, Suita 565-0871, Japan

Contents

Supplementary Methods

Supplementary Table S1, S2

Supplementary Figure S1, S2, S3, S4

Supplementary References

Supplementary Methods

Strain constructions used in Supplementary Information

S. pombe strains expressing *hta2* mutants—namely, *hta2*(*R18A*), *hta2*(*R18A K21A*), *hta2*(*K13A R18A K21A*), *hta2*(*S121A*), and *hta2*(*S127A*)—from the *lys1* locus were constructed as follows: First, the *hta2* gene fragment containing its promoter (from –561 nt), coding region, and terminator (603 nt after the stop codon) was ligated into plasmid pHSG299 (TaKaRa Bio). Next, the resulting plasmid, pHSG-hta2, was used as a template for PCR-based site-directed mutagenesis. The original and mutagenized codons in each *hta2* mutant were as follows: AAG was changed to GCG in *hta2*(*K13A*); CGT was changed to GCT in *hta2*(*R18A*); AAG was changed to GCG in *hta2*(*K21A*); TCT was changed to GCT in *hta2*(*S121A*); AGC was changed to GCC in *hta2*(*S127A*). The mutations were confirmed by sequencing. The resulting plasmids, namely, pHSG-hta2(R18A), pHSG-hta2(R18A K21A), pHSG-hta2(K13A R18A K21A), pHSG-hta2(S121A), and pHSG-hta2(S127A), were digested and ligated into the integration vector pYC36. The resulting plasmids, namely, pYC36-hta2(R18A), pYC36-hta2(R18A K21A), pYC36-hta2(K13A R18A K21A), pYC36-hta2(S121A), and pYC36-hta2(S127A), were integrated into the chromosome at the *lys1* locus. Integration was confirmed by PCR.

Because we could not isolate the integrants of pYC36-hta2(R18A K21A) or pYC36-hta2(K13A R18A K21A) in the Δ*hta2* background by direct integration or by integrating in the WT background first and then backcrossing with the Δ*hta2* background, *hta2*(*R18A K21A*) or *hta2*(*K13A R18A K21A*) expression must be toxic, causing lethality in the Δ*hta2* background.

S. pombe strains expressing *hta2* mutants fused to the FLAG-tag and *mei4DSR*—namely, *hta2-FLAG*, *hta2-FLAG:mei4DSR*, *hta2*(*R18A K21A*)*-FLAG:mei4DSR*, and *hta2*(*K13A R18A K21A*)*-FLAG:mei4DSR*—from the *lys1* locus were constructed as follows: First, *mei4DSR*[49] was used to repress *hta2* mutant expression in vegetative cells; the FLAG-tag was also used to check *hta2* mutant expression. Next, the FLAG coding sequence GATTACAAGGACGACGATGACAAG was added just before the stop codon of *hta2* in plasmid pHSG-hta2, pHSG-hta2(R18A K21A), or pHSG-hta2(K13A R18A K21A) by inverse PCR; the resulting plasmids were pHSG-hta2-FLAG, pHSG-hta2(R18A K21A)-FLAG, and pHSG-hta2(K13A R18A K21A)-FLAG. Then, *mei4DSR* (486–828) was added just after the stop codon of *hta2* in plasmid pHSG-hta2-FLAG, pHSG-hta2(R18A K21A)-FLAG, or pHSG-hta2(K13A R18A

K21A)-FLAG using the In-Fusion HD Cloning Kit (TaKaRa Bio). The resulting plasmids, namely, pHSG-hta2-FLAG:mei4DSR, pHSG-hta2(R18A K21A)-FLAG:mei4DSR, and pHSG-hta2(K13A R18A K21A)-FLAG:mei4DSR, and plasmid pHSG-hta2-FLAG were digested and ligated into the integration vector pYC36. Finally, the plasmids formed, namely, pYC36-hta2-FLAG:mei4DSR, pYC36-hta2(R18A K21A)-FLAG:mei4DSR, pYC36-hta2(K13A R18A K21A)-FLAG:mei4DSR, and pYC36-hta2-FLAG, were integrated into the chromosome at the *lys1* locus. Integration was confirmed by PCR.

S. pombe strain deleted of *dbl2* gene (Δ*dbl2*) or expressing Nuc1-GFP (not Nuc1-GFP-3HA) was constructed using PCR-based gene targeting[35,36]. Gene deletion or gene tagging was confirmed by PCR and sequencing.

In this study, marker switches[36] were performed to make *nuc1-GFP-3HA:hygR* and *nuc1-mCherry:natR* from *nuc1-GFP-3HA:kanR*[50] and *nuc1-mCherry:kanR*[12], respectively.

Thiolutin treatment

Meiotic cells prepared on EMM2-N plate were suspended in liquid EMM2-N containing 0–5 μg/ml thiolutin and sonicated for a few seconds. Cell suspension was transferred to glass-bottomed culture dishes coated with 0.2 mg/mL of soybean lectin and observed under a microscope.

Supplementary Table S1. *S. pombe* strains used in this study.

Strain	Genotype
TGO350	h^{90} *lys1:(Pnda3:GFP-NLS)*
TGO351	h^{90} *lys1:(Pnda3:GFP-NLS) Δhta1::hygR*
TGO352	h^{90} *lys1:(Pnda3:GFP-NLS) Δhta2::hygR*
TGO485	h^{90} *lys1:pYC36 nuc1-GFP-3HA:kanR Δhta2::hygR*
TGO487	h^{90} *lys1:hta2 nuc1-GFP-3HA:kanR Δhta2::hygR*
TGO542	h^{90} *ura4 lys1:(Pnda3:GFP-NLS) Δcds1::ura4*
TGO543	h^{90} *ura4 lys1:(Pnda3:GFP-NLS) Δcds1::ura4 Δhta2::hygR*
TGO647	h^{90} *lys1:(Pnda3:GFP-NLS) aur1R:(Pnda3:GFP-atb2)*
TGO648	h^{90} *lys1:(Pnda3:GFP-NLS) aur1R:(Pnda3:GFP-atb2) Δhta2::hygR*
TGO728	h^{90} *lys1:htb1 htb1-GFP*
TGO729	h^{90} *lys1:htb1 htb1-GFP Δhta2::hygR*
CT2121-4	h^{90} *leu1 ura4 lys1 ade6-216 nhe1:(kanR:ura4:lacOP) his7:(Pdis1:GFP-lacI-NLS)*
TGO462	h^{90} *leu1 ura4 lys1 ade6-216 nhe1:(kanR:ura4:lacOP) his7:(Pdis1:GFP-lacI-NLS) Δhta2::hygR*
YW537	h^{90} *leu1 ura4 lys1 ade6-216 B1:(kanR:ura4:lacOP) his7:(Pdis1:GFP-lacI-NLS)*
TGO463	h^{90} *leu1 ura4 lys1 ade6-216 B1:(kanR:ura4:lacOP) his7:(Pdis1:GFP-lacI-NLS) Δhta2::hygR*
TGO566	h^{90} *leu1 ura4 lys1 ade6-210 rDNA:5xlacOP his7:(Pdis1:GFP-lacI-NLS) nuc1-mCherry:kanR*
TGO578	h^{90} *leu1 ura4 lys1 ade6-210 rDNA:5xlacOP his7:(Pdis1:GFP-lacI-NLS) nuc1-mCherry:kanR Δhta2::hygR*
TGO443	h^{90} *nuc1-GFP-3HA:kanR*
TGO444	h^{90} *nuc1-GFP-3HA:kanR Δhta2::hygR*
TGO804	h^{90} *hta1-GFP*
TGO808	h^{90} *hta2-GFP*
TGO629	h^{90} *leu1:pYC28 lys1:pYC36 aur1R:pYC33 nuc1-GFP-3HA:kanR Δhta2::hygR*
TGO630	h^{90} *leu1:pYC28 lys1:hta1 aur1R:pYC33 nuc1-GFP-3HA:kanR Δhta2::hygR*
TGO631	h^{90} *leu1:hta1 lys1:hta1 aur1R:pYC33 nuc1-GFP-3HA:kanR Δhta2::hygR*
TGO632	h^{90} *leu1:hta1 lys1:hta1 aur1R:hta1 nuc1-GFP-3HA:kanR Δhta2::hygR*
TGO399	h^{90} *lys1:(Pnda3:GFP-NLS) hta2::hta1*
TGO477	h^{90} *nuc1-GFP-3HA:hygR*
TGO521	h^{90} *ura4 Δhta2::ura4 nuc1-GFP-3HA:hygR*
TGO522	h^{90} *Δ(hhf1 hht1)::kanR Δ(hht3 hhf3)::natR nuc1-GFP-3HA:hygR*
TGO523	h^{90} *ura4 Δhta2::ura4 Δ(hhf1 hht1)::kanR Δ(hht3 hhf3)::natR nuc1-GFP-3HA:hygR*
TGO525	h^{90} *ura4 Δhta2::ura4 Δ(hhf1 hht1)::kanR nuc1-GFP-3HA:hygR*
TGO526	h^{90} *ura4 Δhta2::ura4 Δ(hht3 hhf3)::natR nuc1-GFP-3HA:hygR*
TGO575	h^{90} *nuc1-GFP-3HA:kanR Δhta1::natR*
TGO572	h^{90} *nuc1-GFP-3HA:kanR Phta2:(hygR:Pnmt1):hta2*
TGO579	h^{90} *nuc1-GFP-3HA:kanR Δhta1::natR Phta2:(hygR:Pnmt1):hta2*
TGO595	h^{90} *lys1:hta2(R18A) nuc1-GFP-3HA:kanR Δhta2::hygR*
TGO596	h^{90} *lys1:hta2(S121A) nuc1-GFP-3HA:kanR Δhta2::hygR*
TGO547	h^{90} *lys1:hta2(S127A) nuc1-GFP-3HA:kanR Δhta2::hygR*
TGO687	h^{90} *lys1:pYC36 nuc1-mCherry:natR Δhta2::hygR*
TGO688	h^{90} *lys1:hta2 nuc1-mCherry:natR Δhta2::hygR*
TGO690	h^{90} *lys1:hta2-FLAG nuc1-mCherry:natR Δhta2::hygR*
TGO726	h^{90} *lys1:hta2-FLAG:mei4DSR nuc1-mCherry:natR Δhta2::hygR*
TGO727	h^{90} *lys1:hta2(R18A K21A)-FLAG:mei4DSR nuc1-mCherry:natR Δhta2::hygR*
TGO784	h^{90} *lys1:hta2(K13A R18A K21A)-FLAG:mei4DSR nuc1-mCherry:natR Δhta2::hygR*
TGO536	h^{90} *rad21-GFP:kanR nuc1-mCherry:natR*
TGO537	h^{90} *rad21-GFP:kanR nuc1-mCherry:natR Δhta2::hygR*

TGO707	*h^90 nuc1-GFP:natR*
TGO708	*h^90 nuc1-GFP:natR* Δ*hta2::hygR*
TGO931	*h^90 ura4 Padh41:rec8-3HA:ura4 nuc1-GFP:natR*
TGO932	*h^90 ura4 Padh41:rec8-3HA:ura4 nuc1-GFP:natR* Δ*hta2::hygR*
TGO933	*h^90 ura4 Padh41:rec8-3HA:ura4 nuc1-GFP:natR* Δ*rad21::kanR*
TGO934	*h^90 ura4 Padh41:rec8-3HA:ura4 nuc1-GFP:natR* Δ*rad21::kanR* Δ*hta2::hygR*
TGO634	*h^90 nuc1-GFP-3HA:hygR* Δ*hta2::natR*
TGO779	*h^90 nuc1-GFP-3HA:hygR* Δ*dbl2::kanR*

References of genotypes are as follows: *nuc1-GFP-3HA:kanR*[50]; Δ*cds1::ura4*[51]; *aur1R:*(*Pnda3:GFP-atb2*)[52]; *nhe1:*(*kanR:ura4:lacOP*)[53]; *his7:*(*Pdis1:GFP-lacI-NLS*)[41]; *nuc1-mCherry:kanR*[12]; Δ*(hhf1 hht1)::kanR* & Δ*(hht3 hhf3)::natR*[54]; *rad21-GFP:kanR*[14]; *Padh41:rec8-3HA:ura4* & Δ*rad21::kanR*[55]. All other constructs were made in this study.

lys1:pYC36, *leu1:pYC28*, and *aur1R:pYC33* are the integration of empty vectors used as controls for *lys1:hta2*/*lys1:hta1*, *leu1:hta1*, and *aur1R:hta1*, respectively.

Supplementary Table S2. Summary of types of test methods and *P*-values in statistical analyses.

Figure	Method	Label	Sample 1	n	Sample 2	n	*P*-value
1C	Tukey's	-	*WT*	3	Δ*hta1*	3	0.9091
1C	Tukey's	-	*WT*	3	Δ*hta2*	3	< 0.0001
1C	Tukey's	-	Δ*hta1*	3	Δ*hta2*	3	< 0.0001
1D	Student's *t*	-	Δ*hta2*	3	Δ*hta2* + *hta2*	3	< 0.0001
2B	Tukey's	horsetail	*WT*	26	Δ*hta2*	33	< 0.0001
2B	Tukey's	horsetail	*WT*	26	Δ*cds1*	44	0.0207
2B	Tukey's	horsetail	*WT*	26	Δ*hta2* Δ*cds1*	45	< 0.0001
2B	Tukey's	horsetail	Δ*hta2*	33	Δ*cds1*	44	< 0.0001
2B	Tukey's	horsetail	Δ*hta2*	33	Δ*hta2* Δ*cds1*	45	0.0031
2B	Tukey's	horsetail	Δ*cds1*	44	Δ*hta2* Δ*cds1*	45	< 0.0001
2D	Student's *t*	HT end – prometa I	*WT*	35	Δ*hta2*	32	0.0044
2D	Student's *t*	prometa I – ana I	*WT*	35	Δ*hta2*	32	0.327
2D	Student's *t*	ana I – prometa II	*WT*	35	Δ*hta2*	32	< 0.0001
2D	Student's *t*	prometa II – ana II	*WT*	35	Δ*hta2*	32	0.0383
3B	Tukey's	-	*WT*	3	Δ*hta1*	3	0.8873
3B	Tukey's	-	*WT*	3	Δ*hta2*	3	< 0.0001
3B	Tukey's	-	Δ*hta1*	3	Δ*hta2*	3	< 0.0001
3C	Student's *t*	-	Δ*hta2*	3	Δ*hta2* + *hta2*	3	< 0.0001
6C	Student's *t*	-	H2Aα-GFP	9	H2Aβ-GFP	11	< 0.0001
6F	Student's *t*	H2B-GFP	*WT*	15	Δ*hta2*	25	< 0.0001
7A	Tukey's	Spores	Δ*hta2*	3	Δ*hta2* + *hta1 ×1*	3	0.0659
7A	Tukey's	Spores	Δ*hta2*	3	Δ*hta2* + *hta1 ×2*	3	< 0.0001
7A	Tukey's	Spores	Δ*hta2*	3	Δ*hta2* + *hta1 ×3*	3	< 0.0001
7A	Tukey's	Spores	Δ*hta2* + *hta1 ×1*	3	Δ*hta2* + *hta1 ×2*	3	< 0.0001
7A	Tukey's	Spores	Δ*hta2* + *hta1 ×1*	3	Δ*hta2* + *hta1 ×3*	3	< 0.0001
7A	Tukey's	Spores	Δ*hta2* + *hta1 ×2*	3	Δ*hta2* + *hta1 ×3*	3	0.0487
7A	Tukey's	Nuclear division at MI	Δ*hta2*	3	Δ*hta2* + *hta1 ×1*	3	0.0051
7A	Tukey's	Nuclear division at MI	Δ*hta2*	3	Δ*hta2* + *hta1 ×2*	3	< 0.0001
7A	Tukey's	Nuclear	Δ*hta2*	3	Δ*hta2*	3	< 0.0001

		division at MI			+ *hta1 ×3*		
7A	Tukey's	Nuclear division at MI	Δ*hta2* + *hta1 ×1*	3	Δ*hta2* + *hta1 ×2*	3	0.001
7A	Tukey's	Nuclear division at MI	Δ*hta2* + *hta1 ×1*	3	Δ*hta2* + *hta1 ×3*	3	0.0009
7A	Tukey's	Nuclear division at MI	Δ*hta2* + *hta1 ×2*	3	Δ*hta2* + *hta1 ×3*	3	0.9998
7B	Tukey's	Spores	*WT*	3	Δ*hta2*	3	< 0.0001
7B	Tukey's	Spores	*WT*	3	*hta2::hta1*	3	0.5526
7B	Tukey's	Spores	Δ*hta2*	3	*hta2::hta1*	3	< 0.0001
7B	Tukey's	Nuclear division at MI	*WT*	3	Δ*hta2*	3	< 0.0001
7B	Tukey's	Nuclear division at MI	*WT*	3	*hta2::hta1*	3	1
7B	Tukey's	Nuclear division at MI	Δ*hta2*	3	*hta2::hta1*	3	< 0.0001

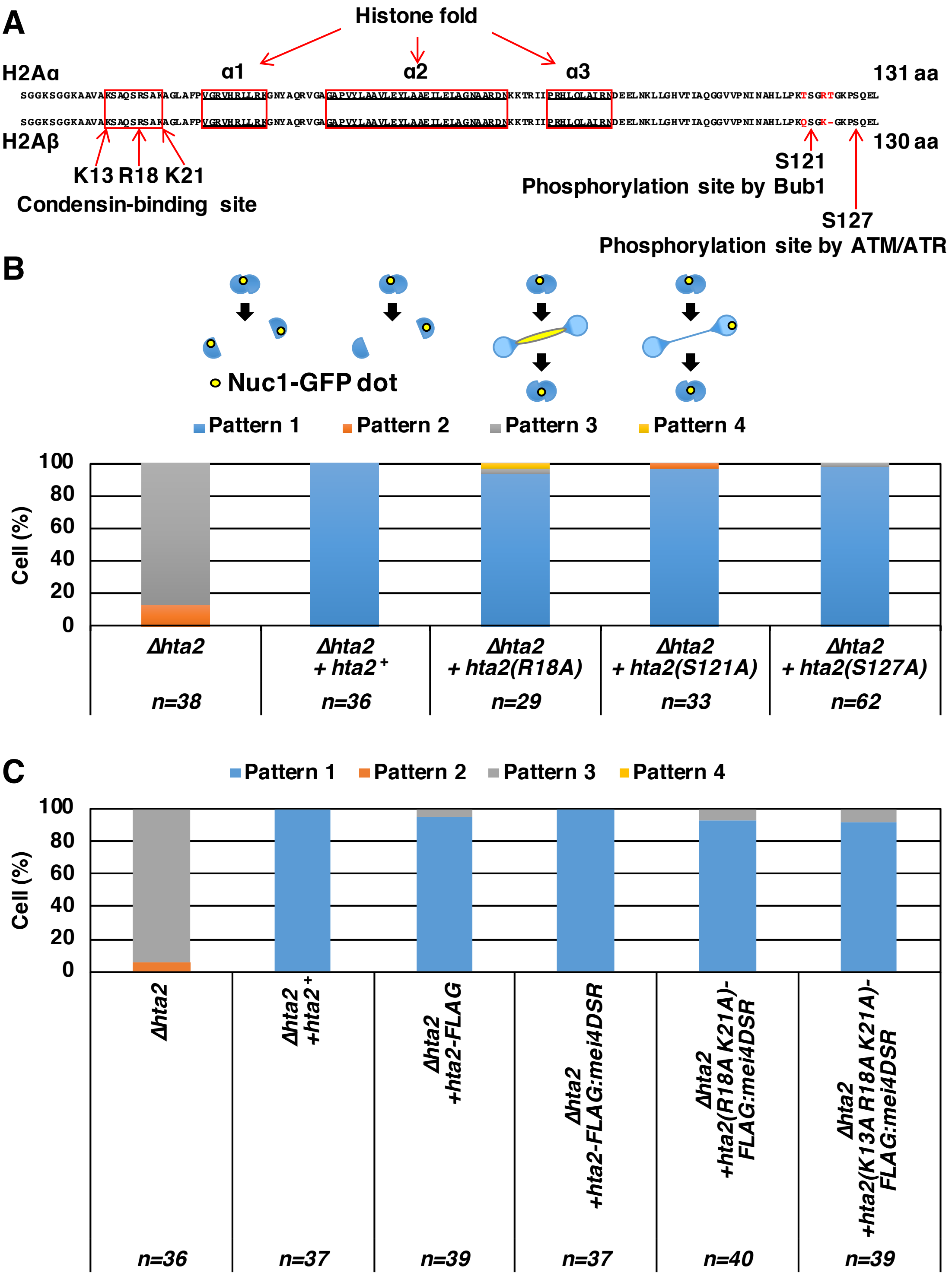

Supplementary Fig. S1. Characterization of the Nuc1 segregation pattern in Δ*hta2* cells expressing *hta2* mutants. (**A**) Alignment of H2Aα and H2Aβ. Red letters: different amino acid residues between H2Aα and H2Aβ; red boxes: histone fold domains (α1, α2, and α3) and the condensin-binding site, including K13, R18, and K21. Phosphorylation sites by Bub1 kinase (S121) and ATM/ATR kinases (S127) are also shown. (**B**) Frequency of cells showing the patterns 1–4 in meiosis I in Δ*hta2* (TGO485) cells or Δ*hta2* cells expressing WT *hta2* (TGO487; "Δ*hta2* + *hta2*$^{+}$"), *hta2*(*R18A*) (TGO595; "Δ*hta2* + *hta2*(*R18A*)"), *hta2*(*S121A*) (TGO596 ; "Δ*hta2* + *hta2*(*S121A*)"), or *hta2*(*S127A*) (TGO547; "Δ*hta2* + *hta2*(*S127A*)"). rDNA was labeled with Nuc1-GFP.

Patterns of segregation were classified into four categories based on nuclear division and Nuc1 segregation, as in Fig**.** 5A: normal nuclear division with divided nucleoli (pattern 1), nuclear division with the nucleolus remaining in one of the divided nuclei (pattern 2), reunion of divided nuclei with the connected nucleolus (pattern 3), and reunion of divided nuclei with the nucleolus remaining in one of the divided nuclei (pattern 4). The number of cells examined is shown at the bottom of the graph. (**C**) Frequency of cells showing the patterns 1–4 in meiosis I in Δ*hta2* (TGO687) cells or Δ*hta2* cells expressing WT *hta2* (TGO688; "Δ*hta2* + *hta2*$^{+}$"), *hta2-FLAG* (TGO690; "Δ*hta2* + *hta2-FLAG*"), *hta2-FLAG:mei4DSR* (TGO726; "Δ*hta2* + *hta2-FLAG:mei4DSR"*), *hta2*(*R18A K21A*)*-FLAG:mei4DSR* (TGO727; "Δ*hta2* + *hta2*(*R18A K21A*)*-FLAG:mei4DSR"*), or *hta2*(*K13A R18A K21A*)*-FLAG:mei4DSR* (TGO784; "Δ*hta2* + *hta2*(*K13A R18A K21A*)*-FLAG:mei4DSR*"). rDNA was labeled with Nuc1-mCherry. Patterns of segregation were classified into four categories as in (**A**). The number of cells examined is shown at the bottom of the graph.

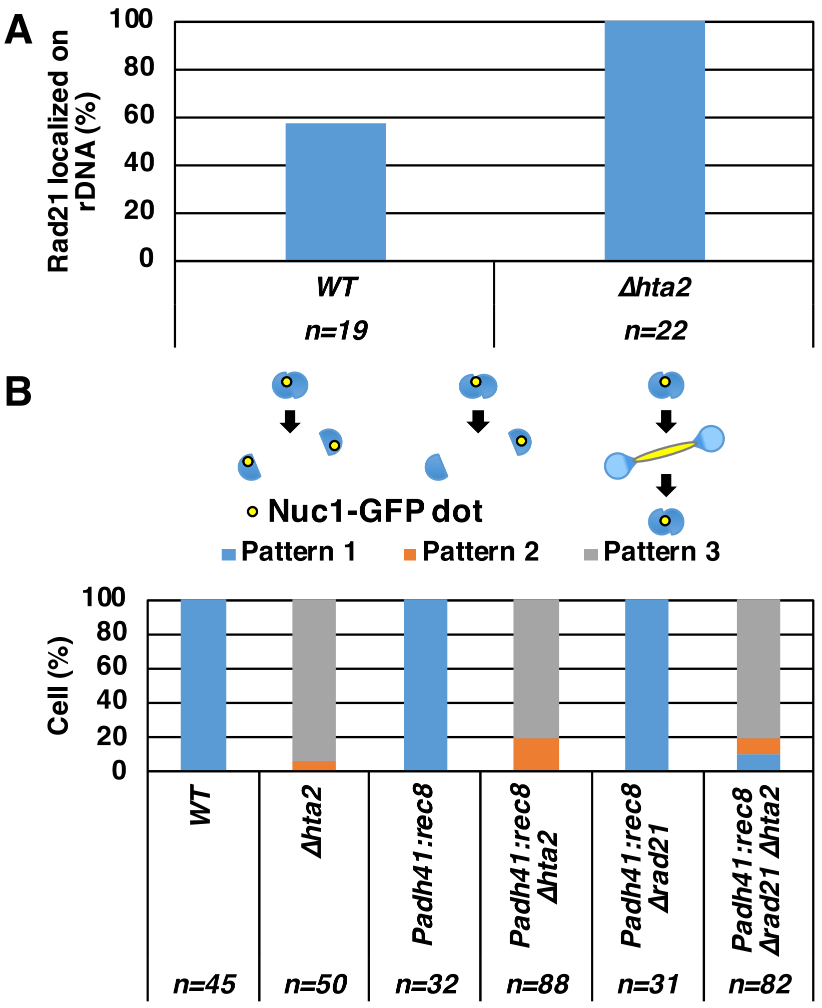

Supplementary Fig. S2. Characterization of the Nuc1 segregation pattern in cohesin mutant cells. (**A**) Frequency of cells showing localization of Rad21-GFP on rDNA during the horsetail stage. rDNA was labeled with Nuc1-mCherry. The number of cells examined is specified at the bottom of the graph. (**B**) Frequency of cells showing patterns 1–3 in meiosis I in WT (TGO707), Δ*hta2* (TGO708), *Padh41:rec8* (TGO931), *Padh41:rec8* Δ*hta2* (TGO932), *Padh41:rec8* Δ*rad21* (TGO933), and *Padh41:rec8* Δ*rad21* Δ*hta2* (TGO934) cells. rDNA was labeled with Nuc1-GFP. Patterns of segregation were classified into three categories based on nuclear division and Nuc1 segregation: normal nuclear division with divided nucleoli (pattern 1), nuclear division with the nucleolus remaining in one of the divided nuclei (pattern 2), and reunion of divided nuclei with the connected nucleolus (pattern 3). The number of cells examined is shown at the bottom of the graph. Note that Δ*rad21* lethality was complemented with ectopic expression of Rec8 from the attenuated *adh1* promoter (*Padh41:rec8*).

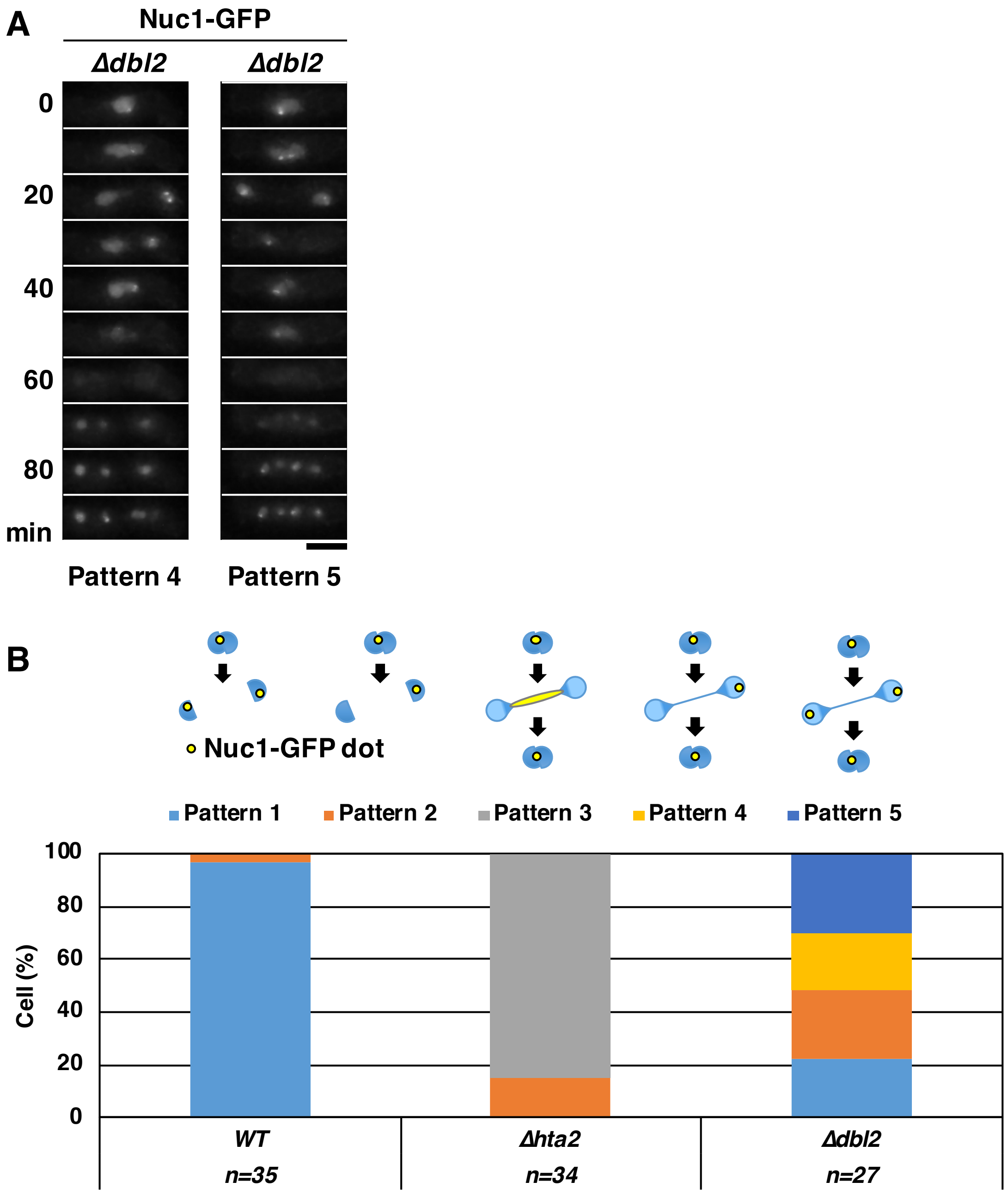

Supplementary Fig. S3. Characterization of Nuc1 segregation pattern in Δ*dbl2* cells. (**A**) Time-lapse images of meiosis I progression in Δ*dbl2* (TGO779) cells. rDNA was labeled with Nuc1-GFP. Patterns of segregation were classified into five categories based on nuclear division and Nuc1 segregation: normal nuclear division with divided nucleoli (pattern 1), nuclear division with the nucleolus remaining in one of the divided nuclei (pattern 2), reunion of divided nuclei with the connected nucleolus (pattern 3), reunion of divided nuclei with the nucleolus remaining in one of the divided nuclei (pattern 4), and reunion of divided nuclei with the divided nucleoli (pattern 5). Only images of patterns 4 and 5 are shown. Numbers indicate the time elapsed after anaphase I onset. Scale bar, 5 µm. (**B**) Frequency of cells showing the patterns 1–5 in meiosis I in WT (TGO477), Δ*hta2* (TGO634), and Δ*dbl2* (TGO779) cells. The number of cells examined is shown at the bottom of the graph. In Δ*dbl2* cells, patterns 4 and 5 were observed, but not pattern 3, suggesting that rDNA was not involved in the reunion of the divided nuclei.

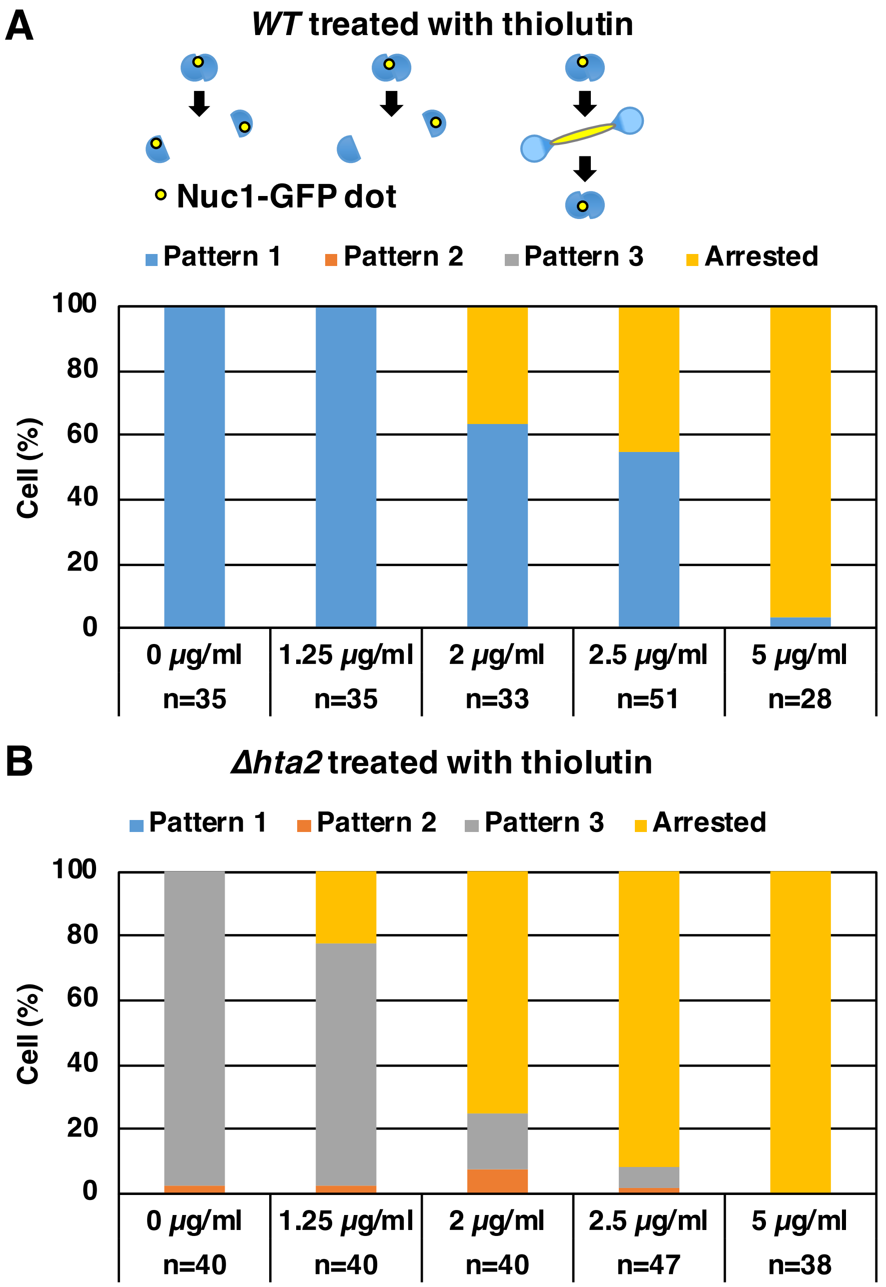

Supplementary Fig. S4. Characterization of the Nuc1 segregation pattern in thiolutin-treated cells. Frequency of cells showing the patterns 1–3 and arrested in meiosis I in WT (TGO443) (**A**) and Δ*hta2* (TGO444) (**B**) cells treated with 0–5 μg/ml thiolutin, an inhibitor of RNA polymerase. rDNA was labeled with Nuc1-GFP. Patterns of segregation were classified into four categories based on nuclear division and Nuc1 segregation: normal nuclear division with divided nucleoli (pattern 1), nuclear division with the nucleolus remaining in one of the divided nuclei (pattern 2), reunion of divided nuclei with the connected nucleolus (pattern 3), and no meiosis I during a 7 h observation (arrested). The number of cells examined is specified at the bottom of the graph. In wild type cells, the frequency of pattern 1 decreased and that of pattern 4 increased with increasing concentrations of thiolutin. In Δ*hta2* cells, the frequency of pattern 3 was predominant but replaced by the frequency of pattern 4 with no increase in pattern 1 as the concentration of thiolutin was increased.

Supplementary References

49. Yamashita, A. *et al*. Hexanucleotide motifs mediate recruitment of the RNA elimination machinery to silent meiotic genes. *Open Biol.* **2,** 120014 (2012).
50. Hayashi, A. *et al*. Localization of gene products using a chromosomally tagged GFP-fusion library in the fission yeast *Schizosaccharomyces pombe*. *Genes Cells* **14,** 217–225 (2009).
51. Murakami, H. & Nurse, P. Meiotic DNA replication checkpoint control in fission yeast. *Genes Dev.* **13,** 2581–2593 (1999).
52. Masuda, H., Fong, C. S., Ohtsuki, C., Haraguchi, T. & Hiraoka, Y. Spatiotemporal regulations of Wee1 at the G2/M transition. *Mol. Biol. Cell* **22,** 555–569 (2011).
53. Ding, D.-Q., Yamamoto, A., Haraguchi, T. & Hiraoka, Y. Dynamics of Homologous Chromosome Pairing during Meiotic Prophase in Fission Yeast. *Dev. Cell* **6,** 329–341 (2004).
54. Takayama, Y. & Takahashi, K. Differential regulation of repeated histone genes during the fission yeast cell cycle. *Nucleic Acids Res*. **35,** 3223–3237 (2007).
55. Sakuno, T., Tada, K. & Watanabe, Y. Kinetochore geometry defined by cohesion within the centromere. *Nature* **458,** 852–858 (2009).
